# Protist communities are more sensitive to nitrogen fertilization than other microorganisms in diverse agricultural soils

**DOI:** 10.1186/s40168-019-0647-0

**Published:** 2019-02-27

**Authors:** Zhi-Bo Zhao, Ji-Zheng He, Stefan Geisen, Li-Li Han, Jun-Tao Wang, Ju-Pei Shen, Wen-Xue Wei, Yun-Ting Fang, Pei-Pei Li, Li-Mei Zhang

**Affiliations:** 1 State Key Laboratory of Urban and Regional Ecology, Research Center for Eco-Environmental Sciences, Chinese Academy of Sciences, Beijing, 100085 China; 2 University of Chinese Academy of Sciences, Beijing, 100049 China; 3 Faculty of Veterinary and Agricultural Sciences, The University of Melbourne, Parkville, VIC 3010 Australia; 4 Department of Terrestrial Ecology, Netherlands Institute of Ecology NIOO-KNAW, 6708 PB Wageningen, The Netherlands; 5 Key Laboratory of Agro-ecological Processes in Subtropical Regions and Taoyuan Station of Agro-Ecology Research, Institute of Subtropical Agriculture, Chinese Academy of Sciences, Changsha, 410125 China; 6 CAS Key Laboratory of Forest Ecology and Management, Institute of Applied Ecology, Chinese Academy of Sciences, Shenyang, 110016 China; 7 College of Resource and Environmental Sciences, Henan Agricultural University, Zhengzhou, 450002 China

**Keywords:** Soil protists, Microbiome, Soil type, Nitrogen fertilizers, High-throughput sequencing

## Abstract

**Background:**

Agricultural food production is at the base of food and fodder, with fertilization having fundamentally and continuously increased crop yield over the last decades. The performance of crops is intimately tied to their microbiome as they together form holobionts. The importance of the microbiome for plant performance is, however, notoriously ignored in agricultural systems as fertilization disconnects the dependency of plants for often plant-beneficial microbial processes. Moreover, we lack a holistic understanding of how fertilization regimes affect the soil microbiome. Here, we examined the effect of a 2-year fertilization regime (no nitrogen fertilization control, nitrogen fertilization, and nitrogen fertilization plus straw amendment) on entire soil microbiomes (bacteria, fungi, and protist) in three common agricultural soil types cropped with maize in two seasons.

**Results:**

We found that the application of nitrogen fertilizers more strongly affected protist than bacterial and fungal communities. Nitrogen fertilization indirectly reduced protist diversity through changing abiotic properties and bacterial and fungal communities which differed between soil types and sampling seasons. Nitrogen fertilizer plus straw amendment had greater effects on soil physicochemical properties and microbiome diversity than nitrogen addition alone. Moreover, nitrogen fertilization, even more together with straw, increased soil microbiome network complexity, suggesting that the application of nitrogen fertilizers tightened soil microbiomes interactions.

**Conclusions:**

Together, our results suggest that protists are the most susceptible microbiome component to the application of nitrogen fertilizers. As protist communities also exhibit the strongest seasonal dynamics, they serve as the most sensitive bioindicators of soil changes. Changes in protist communities might have long-term effects if some of the key protist hubs that govern microbiome complexities as top microbiome predators are altered. This study serves as the stepping stone to promote protists as promising agents in targeted microbiome engineering to help in reducing the dependency on exogenous unsustainably high fertilization and pesticide applications.

**Electronic supplementary material:**

The online version of this article (10.1186/s40168-019-0647-0) contains supplementary material, which is available to authorized users.

## Background

Soils provide the basis of our life. We not only live on soils, but soils are also needed for crop production that forms the base of most of our food [1]. While the importance of the abiotic part of soils including soil water and nutrient levels is widely acknowledged, the role of soil biota has received less attention. In fact, soils serve as the habitat of arguably most of the taxonomic biodiversity on planet Earth. We are slowly beginning to decipher this vast biodiversity and to understand the functional importance of the crop-associated microbiome especially in the plant-influenced rhizosphere in promoting plant growth, increasing plant tolerance, and protecting plants against pathogens [2–7]. Despite the acknowledged importance of the soil biodiversity in sustaining soil fertility and controlling plant performance, the positive functional role of soil biodiversity especially in agricultural systems is often ignored or treated as predominantly being plant deleterious that needs to be controlled. Artificial pesticides and fertilizers have disconnected our dependency on positive services soil biodiversity provides for plant performance, but long-term pesticide and fertilizer applications are increasingly shown to be unsustainable [8]. To increase sustainability, we first need a better understanding of the processes induced by agricultural managements on soil biodiversity.

Nitrogen fertilization is a commonly used agricultural practice to increase crop production [9]. However, the intensive application can lead to soil acidification, greenhouse gas N_2_O emission, nitrate leaching [10, 11], and biodiversity losses [12, 13]. Biodiversity decreases subsequently affect nitrogen cycling and soil multifunctionality [2, 14, 15]. As a traditional agronomic practice to improve soil fertility, straw retention showed significant effects on improving soil organic carbon contents and at reducing nitrogen loss [16, 17]. The application of combined organic and chemical nitrogen fertilizers is thus encouraged in modern sustainable and environmental-friendly agriculture. However, to date, the effects of nitrogen addition alone or in combination with straw incorporation on entire soil microbiomes are not understood in detail.

Most work examining the impact of fertilization on microbiome composition and function has focused on bacterial and fungal communities. Their community diversity was shown to be reduced by long-term nitrogen fertilizer application [9, 13, 18]. Protists as another microbial group have received little attention despite their key role in controlling bacterial and fungal populations [19]. Previous morphogroup-based studies focusing on amoebae have shown that both nitrogen addition and plant residue amendment could affect protist density, while plant residue did not influence the community structure of protists [20, 21]. However, the taxonomic resolution of these morphogroup-approaches is low, with individual morphogroups consisting of phylogenetically and functionally diverse taxa [19] and might therefore undermine important ecological differences. High-throughput sequencing approaches now enable a deeper resolution of protist communities and have revealed that long-term mineral fertilization reduced protist diversity in soils compared with organic fertilization [22]. In contrast, Lentendu et al. [23] reported that organic fertilizers more strongly altered protist communities than mineral fertilizers. The difference between these studies might have originated from a focus on single soil types and sampling points as protist communities are structured by abiotic conditions [24–28] that often differ between soil types and can vary over sampling times [29]. Therefore, we miss a cumulative understanding of how fertilization, particularly widely applied nitrogen amendments, affects soil protists. Furthermore, to better understand the entire microbiome responses to fertilizer application and to disentangle the role of key microbial taxa in microbiome communities after fertilization, an integrative study of all microbial groups is needed.

We here performed a holistic analysis of soil microbiome members including bacteria, fungi, and protists using group-specific high-throughput sequencing approaches in three major agricultural crop production areas, corresponding to three major soil types, in China. These soils were planted with the same crop species (*Zea mays*) and treated with a consistent fertilization regime over 2 years. The microbiome structure was analyzed in summer and autumn. Our first hypothesis was that the application of nitrogen fertilizers reduces soil microbial diversity, with more pronounced effects on first trophic level bacteria and fungi than the predominantly predatory protists. Furthermore, we expected that straw amendments could mitigate the negative nitrogen-fertilizer-induced effects on biodiversity (hypothesis 2). We also tested whether these effects differ between soil types and sampling seasons. We analyzed diverse abiotic parameters to disentangle the importance of abiotic and biotic factors regulating microbiome interactions through structural equation models (SEM) and network analyses.

## Results

### Soil physicochemical properties

Soil physicochemical properties were significantly different between soil type (*P* = 0.001, *R*^2^ = 0.659) and season (*P* = 0.001, *R*^2^ = 0.096; Table 1). The black and red soils were acidic, with a mean pH value at 5.14 and 4.66, respectively, whereas the fluvo-aquic soil was weakly alkaline with a mean pH value at 7.76 (Additional file 1: Table S2). Two-year nitrogen fertilizations further decreased soil pH in the black and red soils (*P* < 0.05) but did not in the fluvo-aquic soil (Additional file 1: Table S2). In summer, the water content of the red soil was highest (22.83 ± 0.88%) among the three soil types (*P* < 0.05; Additional file 1: Table S2). In addition, the red soil had a lower C/N ratio (9.06 ± 0.20) than the black (11.76 ± 0.63) and fluvo-aquic soils (13.36 ± 0.82, *P* < 0.05; Additional file 1: Table S2). Compared to summer, the moisture of the black and fluvo-aquic soils increased in autumn (Additional file 1: Table S2). The concentration of NO_3_^−^-N of each treatment in autumn was lower than that in summer (*P* < 0.05).

**Table 1:** The effects of soil type, season, and fertilization treatment on the differentiations of soil physicochemical properties and bacterial, fungal, and protist communities based on PERMANOVA

		Fertilization	Soil type	Season	Fertilization × soil type	Fertilization × season	Soil type × season	Fertilization × soil type × season
Physicochemical properties	*R* ^2^	0.063	0.659	0.096	0.013	0.008	0.045	0.012
	*P*	0.001	0.001	0.001	0.399	0.271	0.002	0.38
Bacterial community	*R* ^2^	0.013	0.512	0.498	0.007	0.003	− 0.043	0.003
	*P*	0.001	0.001	0.001	0.001	0.002	1	0.004
Fungal community	*R* ^2^	0.017	0.369	0.553	0.009	0.007	0.028	0.005
	*P*	0.001	0.001	0.001	0.001	0.002	0.001	0.004
Protist community	*R* ^2^	0.022	0.310	0.568	0.008	0.009	0.049	0.009
	*P*	0.001	0.001	0.001	0.020	0.003	0.001	0.011

The application of nitrogen fertilizers (N and NS treatments) significantly influenced the overall soil physicochemical properties (synthesized by pH, moisture, C/N ratio, organic matter, dissolved organic carbon, total content of carbon and nitrogen, ammonium nitrogen, and nitrate nitrogen; *P* = 0.001, *R*^2^ = 0.063; Table 1). The N and NS treatments increased soil NH_4_^+^-N and NO_3_^−^-N (Additional file 1: Table S2). However, the effects of fertilization on abiotics differed between soil types. In comparison to the control treatment, the NS treatment significantly decreased pH (*P* < 0.05) in the black and red soils (Additional file 1: Table S2). In addition, the NS treatment significantly increased NO_3_^−^-N (*P* < 0.05) in the red soil (Additional file 1: Table S2). The effects of fertilization also differed between seasons. In comparison to the control treatment, the N treatment significantly (*P* < 0.05) decreased pH in the red soil in summer and in the black soil in autumn (Additional file 1: Table S2). The N treatment significantly increased NO_3_^−^-N (*P* < 0.05) in the black and red soils in summer and increased NH_4_^+^-N (*P* < 0.05) in the black soil in autumn (Additional file 1: Table S2). The NS treatment significantly increased NH_4_^+^-N (*P* < 0.05) in the fluvo-aquic soil in summer and in the black and red soils in autumn (Additional file 1: Table S2). Generally, the NS treatment induced the strongest changes in soil physicochemical properties (Additional file 1: Table S2) such as leading to higher contents of NH_4_^+^-N and NO_3_^−^-N, while reducing pH in soil (Table 3).

### Soil microbiomes

#### Bacterial community

**Fig. 1:**
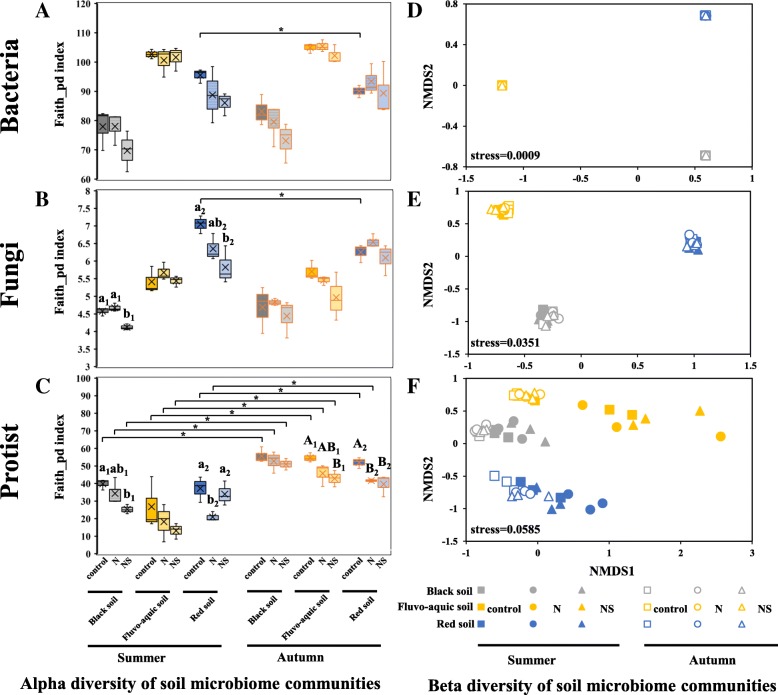
Alpha and beta diversity of the soil bacterial (**A**, **D**), fungal (**B**, **E**), and protist (**C**, **F**) community. Faith_pd index was calculated based on phylogenetic distance at OTU level and displayed in boxplot. The difference in alpha diversity among nitrogen fertilization treatments within a soil type in a season was tested by one-way ANOVA (*P* < 0.05), and only significant differences observed in a comparison group was labeled with letter. Lowercase letter and capital letter represent the groups in summer and autumn, respectively, and the digit behind the letter means different comparison groups. The asterisk labeled implies the significant difference (*P* < 0.05) of alpha diversity observed in the treatment between summer and autumn. Beta diversity was analyzed by nonmetric multidimensional scaling (NMDS) based on unweighted unifrac phylogenetic distance metrics at OTU level and displayed in scatter diagram

After quality filtering, the remaining 2,233,386 sequences were clustered into 14,742 bacterial OTUs. Nitrogen fertilizer treatments did not induce significant (*P* > 0.05) changes in the alpha (phylogenetic, faith_pd index) diversity of bacterial community in the three types of soils (Fig. 1A, Tables 2 and 3). Furthermore, there were no overall differences (*P* > 0.05) in the alpha diversity of bacterial community between summer and autumn, except for the control treatment (*P* < 0.05) in the red soil (Fig. 1A). The beta diversity of bacterial communities showed dramatic variations among the three soil types (Fig. 1D). Soil type (*R*^2^ = 0.512) much more strongly affected bacterial community composition than nitrogen fertilization treatments (*R*^2^ = 0.013; Table 1). The LEfSe analysis revealed that 71 biomarkers affiliating with 10 phyla were sensitive to nitrogen fertilizer treatments in soils (*P* < 0.05, LDA > 2.0; Additional file 1: Figure S2, Table S3). These biomarkers accounted for 2.67% of all taxa retrieved. For instance, five taxa within the order *Xanthomonadales* were more sensitive to nitrogen fertilization, while the genus *Dyella* within this order was significantly enriched in the NS treatment in the black and fluvo-aquic soils in autumn (Additional file 1: Figure S2, Table S3). Four taxa within the order *Rhizobiales* and one genus within the order *Burkholderiales* were susceptible to nitrogen fertilizers, and the genus *Labrys* and an unclassified genus within the order *Burkholderiales* were enriched in the NS treatment in the black soil in autumn (Additional file 1: Figure S2, Table S3).

**Table 2:** The effects of soil type, season, and fertilization treatment on the changes of alpha diversity of bacterial, fungal, and protist communities based on linear mixed model (LMM)

Alpha diversity		Fertilization	Soil type	Season	Fertilization × soil type	Fertilization × season	Soil type × season	Fertilization × soil type × season
Bacterial community	*F*	4.473	98.300	2.188	0.757	0.327	0.242	0.754
	*P*	0.065	< 0.001	0.150	0.561	0.724	0.787	0.563
Fungal community	*F*	7.056	117.140	0.0305	0.565	0.367	1.220	2.942
	*P*	0.027	< 0.001	0.862	0.690	0.696	0.309	0.037
Protist community	*F*	13.163	10.253	142.357	1.536	0.218	5.969	1.475
	*P*	0.006	< 0.001	< 0.001	0.217	0.805	0.007	0.235

With the plot position serial number (*i* _ *j*, where *i* and *j* are the row and column number of the plot, respectively) in the field as a random effect

**Table 3:** Overview of the significance difference based on one-way ANOVA for the effect of nitrogen fertilizers on soil physicochemical properties and alpha diversity of microbiomes in comparison to the control treatment

	Summer			Autumn		
	Black soil	Fluvo-aquic soil	Red soil	Black soil	Fluvo-aquic soil	Red soil
pH	NS	–	N, NS	N, NS	–	NS
Moisture	N	–	–	–	–	–
C/N ratio	N, NS	–	–	–	–	–
OM	–	–	–	–	N	–
DOC	–	–	–	N, NS	–	–
TC	–	–	–	–	–	–
TN	–	–	–	NS	–	–
NH_4_^+^-N	–	NS	–	N, NS	–	NS
NO_3_^−^-N	N, NS	–	N, NS	–	–	NS
Bacterial diversity	–	–	–	–	–	–
Fungal diversity	NS	–	NS	–	–	–
Protist diversity	NS	–	N	–	NS	N, NS

Statistical differences were considered significant at *P* < 0.05

*Abbreviation*: *OM* organic matter, *TC* total content of carbon, *TN* total content of nitrogen, *C/N* the ratio of TC and TN, *Moisture* soil water content, *DOC* dissolved organic carbon, *Control* fertilization treatments including no nitrogen addition, *N* nitrogen addition, *NS* nitrogen + straw addition

#### Fungal community

After quality filtering, the remaining 1,998,108 sequences were clustered into 519 fungal OTUs. The alpha diversity of fungal communities was not affected (*P* > 0.05) by the application of nitrogen fertilizers (N and NS treatments), except in the black and red soils in summer, in which the NS treatment (*P* < 0.05) significantly suppressed the alpha diversity of fungal communities (Fig. 1B, Table 3). Furthermore, there was no difference (*P* > 0.05) in the alpha diversity of fungal community between summer and autumn, except for the control treatment (*P* < 0.05) in the red soil (Fig. 1B). Similar to bacterial community, the application of nitrogen fertilizers (*R*^2^ = 0.017) had weaker effects than soil types (*R*^2^ = 0.369) on fungal community composition and diversity (Tables 1 and 2, Fig. 1E). LEfSe analysis revealed that 51 biomarkers affiliating with five phyla were sensitive to nitrogen fertilizer treatments in soils (*P* < 0.05, LDA > 2.0; Additional file 1: Figure S3, Table S4). These biomarkers were equal to 9.53% of all taxa retrieved. Unlike bacteria, the relative abundance of fungal taxa significantly changed (*P* < 0.05, LDA > 2.0) at a higher taxonomic level under nitrogen fertilization treatments. The phylum *Ascomycota* was significantly increased in the NS treatment while the phyla *Glomeromycota* and *Chytridiomycota* significantly decreased in autumn in the N and NS treatments in the black and fluvo-aquic soil, respectively (Additional file 1: Figure S3, Table S4).

#### Protist community

**Fig. 2:**
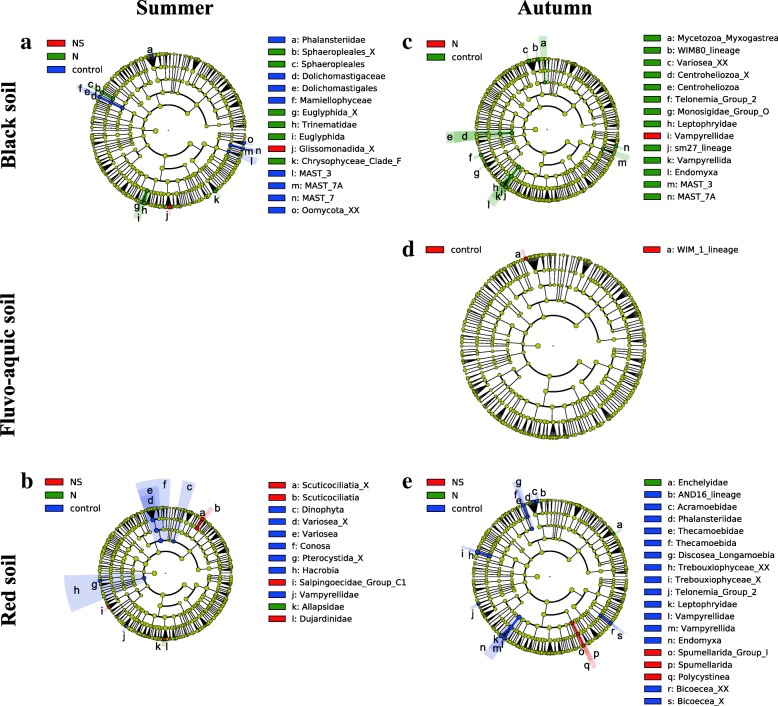
**A**–**E** LEfSe results revealed protist biomarkers (from supergroup level to family level) that were sensitive to nitrogen fertilizers (no nitrogen addition (control) or nitrogen addition (N) or nitrogen + straw addition (NS)) in the black soil in summer (**a**) and autumn (**c**), in the fluvo-aquic soil in autumn (**d**) and in the red soil in summer (**b**) and autumn (**e**). There are five circular rings in the cladogram, each circular ring deposit all taxa within a taxonomic level; the circular ring from inside to outside represents supergroup, phylum, class, order, and family, respectively. The node on the circular ring represents a taxon affiliating within the taxonomic level. Taxa that had significantly higher relative abundance in a certain treatment within each soil type were color-coded within the cladogram according to the Protist Ribosomal Reference (PR2) taxonomy. Soil samplings were conducted in summer and autumn after 2-year fertilizers application. _X represents unidentified lower taxonomic ranks within the respective category

After quality filtering and removal of non-protist eukaryotes, the remaining 454,091 sequences were clustered into 3287 protist OTUs. The application of nitrogen fertilizers (N and NS treatments) more strongly affected protist communities composition (*R*^2^ = 0.022, *P* = 0.001) than bacterial (*R*^2^ = 0.013, *P* = 0.001) and fungal (*R*^2^ = 0.017, *P* = 0.001) communities in soils (Table 1). The application of nitrogen fertilizers also had a greater effect on alpha diversity of protist community (*F* = 13.163, *P* = 0.006) than bacterial (*F* = 7.056, *P* = 0.027) and fungal (*F* = 4.473, *P* = 0.065) communities in soils (Table 2). In general, the application of nitrogen fertilizers decreased the alpha diversity of protists in soils, while the suppression effects (*P* < 0.05) varied among soil types, seasons, and between the N and NS treatments (Fig. 1C, Table 3). The beta diversity of protist communities was, in comparison to bacterial and fungal communities, strongly affected by soil type and the application of nitrogen fertilizers (N and NS treatments) that depended on season (Fig. 1F, Additional file 1: Figure S5). The alpha diversity of protist communities was most strongly affected in the NS treatment (*P* < 0.05; Table 3). Protist community composition and alpha diversity also changed more strongly over season than bacterial and fungal communities (Tables 1 and 2). The alpha diversity of protist communities was significantly higher in autumn than in summer (*P* < 0.05), except for the NS treatment (*P* > 0.05) in the red soil (Fig. 1C). Overall, the alpha diversity of protist communities was positively correlated with moisture (*R* = 0.374, *P* < 0.01) and negatively with NO_3_^−^-N (*R* = − 0.600, *P* < 0.01; Additional file 1: Table S6). LEfSe analysis revealed that 61 biomarkers affiliating with 12 phyla were sensitive to nitrogen fertilizer treatments in soils (*P* < 0.05, LDA > 2.0; Fig. 2, Additional file 1: Table S5). These biomarkers were equal to 11.40% of all taxa retrieved. For instance, protist taxa in the class *Endomyxa*, especially in the endomyxan order *Vampyrellida*, were significantly reduced in the N and NS treatments in the black and the red soils in autumn (Fig. 2, Additional file 1: Table S5). The order *Euglyphida* was significantly enriched by the N treatment in the black soil in summer (Fig. 2, Additional file 1: Table S5).

### Co-occurrence between soil microbiomes

**Fig. 3:**
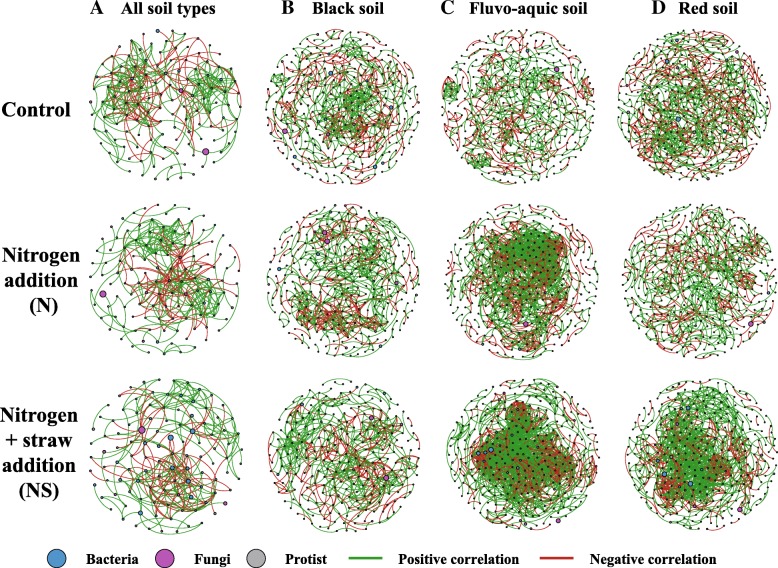
The networks visualize fertilization treatment (including no nitrogen addition (control), nitrogen addition (N), nitrogen + straw addition (NS)) effects on the co-occurrence pattern between protist, bacterial, and fungal taxa at family level in soils. The networks in (**a**) were constructed based on fertilization treatment of all soil types. The networks in (**b**–**d**) were constructed based on fertilization treatment for each soil type. The node size is proportional to the abundance of taxa, and the nodes filled in blue are bacterial taxa, in pink are fungal taxa, and in gray are protist taxa. The edges are colored according to interaction types; positive correlations are labeled with green and negative correlations are coloured in red

In order to determine the general effects of nitrogen fertilization treatments on soil microbiome associations, three networks were constructed for three fertilization practices (control, N, and NS) by combining all microbiomes originating from the three soil types and the two seasons (Fig. 3a). Compared to the control treatment, the clustering coefficient of the network of the N and the NS treatment was increased by 0.010 and 0.005 (Fig. 3a, Table 4), respectively, indicating that soil microbiome associations were more tightened under nitrogen fertilizations. The percentage of protist nodes in the network of the N and the NS treatment was reduced by 2.27 and 3.51%, respectively, in comparison with the control treatment (Fig. 3a, Table 4). Similarly, specific networks focusing on each soil type were created (Fig. 3(b–d), Additional file 1: Figure S4), as soil type most strongly drives microbiome composition (Fig. 1(d–f), Table 1).

**Table 4:** Topological indices of each network in Fig. 3

	All soil types			Black soil			Fluvo-aquic soil			Red soil		
	Control	N	NS	Control	N	NS	Control	N	NS	Control	N	NS
Clustering coefficient	0.339	0.349	0.344	0.382	0.339	0.432	0.415	0.410	0.496	0.361	0.371	0.427
Network density	0.043	0.040	0.041	0.011	0.014	0.018	0.009	0.020	0.030	0.009	0.009	0.022
Number of nodes	124	121	110	343	313	254	368	354	328	409	365	328
Number of edges	325	289	248	648	668	571	637	1236	1635	754	604	1178
Protist nodes (%)	8.06	5.79	4.55	21.87	19.81	16.14	10.05	5.65	6.10	17.11	13.15	14.33
Fungal nodes (%)	21.77	21.49	12.73	19.24	22.04	19.69	18.47	17.80	17.68	20.54	21.92	20.12
Bacterial nodes (%)	70.16	72.73	82.73	58.89	58.15	64.17	71.47	76.55	76.22	62.35	64.93	65.55
Edges linking protist to bacteria (%)	2.77	0	0.40	16.82	17.07	12.78	7.85	4.05	0.43	16.45	12.42	4.24
Edges linking protist to fungi (%)	0.31	0.35	0	6.48	6.74	8.06	3.14	0.49	0.24	6.90	5.46	2.29
Edges linking bacteria to fungi (%)	15.69	14.53	13.31	13.12	17.07	11.56	21.19	10.28	4.46	16.98	24.34	14.35

*Abbreviation*: *Control* no nitrogen addition, *N* nitrogen addition, *NS* nitrogen + straw addition, *Protist nodes (%)* percentage of nodes assigned to protist taxa, *Edges linking protist to bacteria (%)* percentage of edges linking protist taxa to bacterial taxa

These focused network analyses revealed that the percentage of protist nodes in the networks of the N and the NS treatment was reduced by 2.06 and 5.73% in the black soil, by 4.4 and 3.95% in the fluvo-aquic soil, and by 3.96 and 2.78% in the red soil, respectively, compared to the networks of the control treatment in each soil type (Fig. 3(B–D), Table 4). The network density of microbiome networks in the N treatment was increased by 0.003 and 0.011 in the black soil and the fluvo-aquic soil, respectively, compared to the control treatment in each soil type. The network density of microbiome network in the NS treatment was 0.018, 0.030, and 0.022 higher than 0.014, 0.020, and 0.009 in the N treatment, in the black soil, the fluvo-aquic soil, and the red soil, respectively, indicating that soil microbiome formed more tightened associations in responding to the application of nitrogen fertilizers with the NS treatment had greater effects (Fig. 3(B–D), Table 4). Key hub analysis further suggested that protist taxa were as important as bacterial and fungal taxa in soil microbiome networks (Additional file 1: Figure S6). The application of nitrogen fertilizers also reduced the connectivity of protist nodes in microbiome networks (Table 5). A protist taxon within the family *Mesofilidae* was most connected in control networks in the fluvo-aquic soil, while it was not linked to any other microbial taxa in the networks of the N and NS treatments (Table 5). A protist taxon within the family *Allapsidae* and one in the class *Trebouxiophyceae* were not only identified as biomarkers sensitive to N and NS treatments (Fig. 2B, E), but also represented the two protist nodes accounting for the highest connections in control networks in the red soils, with a reduced connectivity in the networks in the N and NS treatments (Table 5). Furthermore, some potential key associations between protist with bacterial and fungal taxa were found as strongly linked edges in these networks (*P* < 0.05). Five edges were common in control treatments and one edge common link in the N treatment in more than one soil type (Additional file 1: Table S7). For instance, predatory protists within the family *Euglyphidae* associated with an unknown *Verrucomicrobia* family in the control treatment in black and fluvo-aquic soils (*P* < 0.05; Additional file 1: Table S7). The protist family *Sandonidae* associated with an unknown fungal family within the order *Chaetothyriales* in the N treatment in black and red soils (*P* < 0.05; Additional file 1: Table S7).

**Table 5:** Protist nodes sensitively responding to nitrogen fertilization in each soil type

Soil type	Node^a^_ID	Control		N		NS		Taxonomic information
		Degree	Rank^b^	Degree	Rank^b^	Degree	Rank^b^	
Black soil	P154	14	4	3	148	0	–	Acanthoecida_X
	P235	11	12	5	103	7	48	Raphid-pennate
Fluvo-aquic soil	P185	10	1	0	–	0	–	Mesofilidae
Red soil	P209	10	3	7	22	2	227	Allapsidae
	P130	10	5	1	302	1	276	Trebouxiophyceae_XX

^a^The nodes represents the top one or two protist nodes with highest degree in the control network within each soil type

^b^The rank is based on the degree order among all microbial nodes in the network

Further results for seasonal dynamics of soil microbiome networks are provided as Additional file 2.

### Influential factors on soil microbiome communities

**Fig. 4:**
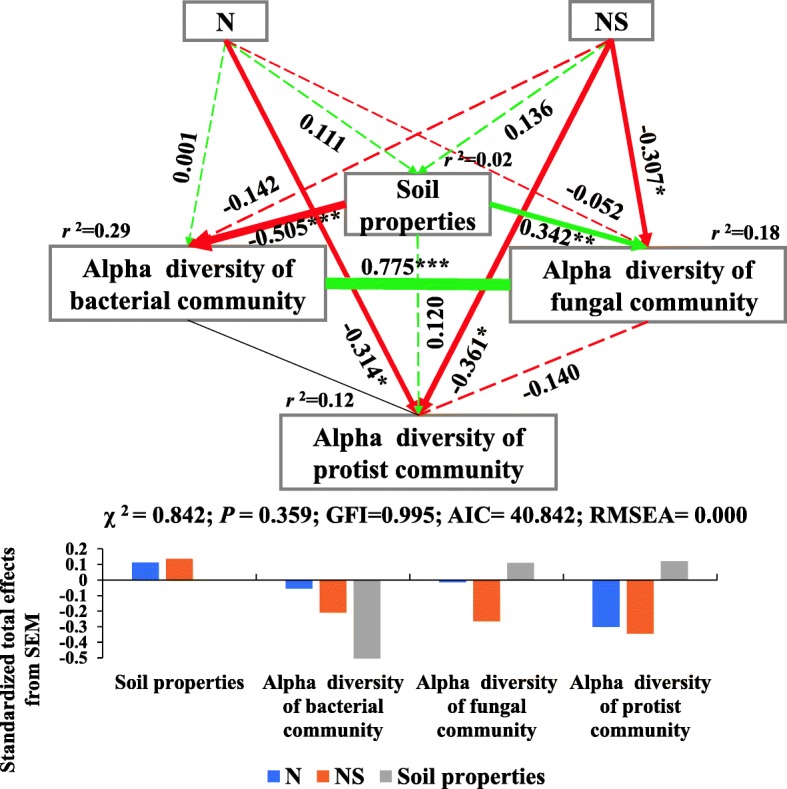
Structural equation model (SEM) illustrating the direct and indirect effects of nitrogen fertilizers (N and NS treatments) on soil physicochemical properties and alpha diversity (faith_pd index) of bacterial, fungal, and protist communities. Continuous and dashed arrows represent the significant and nonsignificant relationships, respectively. Adjacent number that are labeled in the same direction as the arrow represents path coefficients, and the width of the arrow is in proportion to the degree of path coefficients. Green and red arrows indicate positive and negative relationships, respectively. *r*^2^ values indicate the proportion of variance explained by each variable. Significance levels are denoted with *P < 0.05, **P < 0.01, ***P < 0.001. Standardized total effects (direct plus indirect effects) calculated by the SEM are displayed below the SEM. The low chi-square (*χ*^2^), nonsignificant probability level (*P* > 0.05), high goodness-of-fit index (GFI > 0.90), low Akaike information criteria (AIC), and low root-mean-square errors of approximation (RMSEA < 0.05) listed below the SEMs indicate that our data matches the hypothetical models

To further characterize the differentiated effects of the N and NS treatment on soil physicochemical properties and alpha diversity of soil microbiomes (Table 3, Additional file 1: Table S3–S5), structural equation model (SEM) was constructed (Fig. 4). Judging from the standard total effects, the NS treatment had greater effects on soil physicochemical properties and alpha diversity of bacterial, fungal, and protist communities than the N treatment, coinciding with the ANOVA analysis (Fig. 1, Table 3). Furthermore, the average values of the N and NS effect on the alpha diversity were 0.133 for bacterial community, 0.139 for fungal community, and 0.323 for protist community, further confirming that protist diversity was more sensitive to nitrogen fertilizations as also indicated by linear mixed models (LMM) and ANOVA analysis (Tables 2 and 3, Fig. 1).

**Fig. 5:**
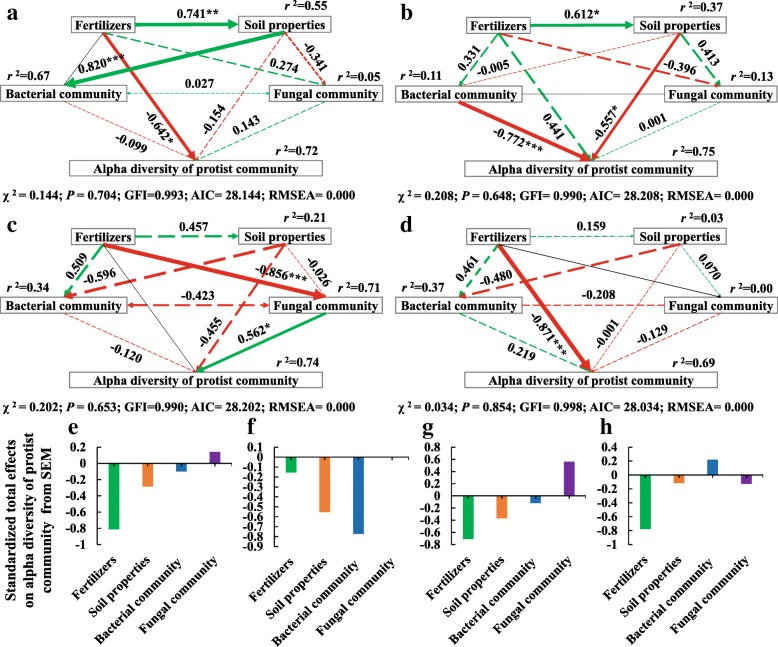
Structural equation model (SEM) illustrating the effects of nitrogen fertilizers, soil physicochemical properties, and bacterial and fungal community on alpha diversity (faith_pd index) of the protist community in the black (**a**) and red soil (**b**) in summer, and in the fluvo-aquic (**c**) and red soil (**d**) in autumn in which protist diversity was significantly reduced. Continuous and dashed arrows represent significant and nonsignificant relationships, respectively. Adjacent numbers that are labeled in the same direction as the arrow are path coefficients, and the width of the arrow is in proportion with the degree of path coefficients. Green and red arrows indicate positive and negative relationships, respectively. *r*^2^ values indicate the proportion of variance explained for each variable. Significant levels are denoted *P < 0.05, **P < 0.01, ***P < 0.001. Standardized total effects (direct plus indirect effects) calculated by the SEMs (**a**, **b**, **c**, **d**) are displayed in **e**, **f**, **g**, **h**, respectively. The low chi-square (*χ*^2^), nonsignificant probability level (*P* > 0.05), high goodness-of-fit index (GFI > 0.90), low Akaike information criteria (AIC), and low root-mean-square errors of approximation (RMSEA < 0.05) listed below the SEMs indicate that our data matches the hypothetical models

SEMs (Fig. 5) further quantified the contribution of each potential influential factor (including nitrogen fertilizers, soil physicochemical properties, bacterial and fungal communities) to the significant reduction (*P* < 0.05) in the alpha diversity (faith_pd index) of protist communities (Fig. 1c, Table 3) induced by nitrogen fertilizers application in different soil types and across seasons. Nitrogen fertilizers were the prominent factor reducing protist diversity in the black soil in summer, in the fluvo-aquic soil in autumn, and in the red soil in autumn (Fig. 5e, g, h). Also, biotic interactions directly affected protist diversity, with the bacterial community contributing more to the reductions in alpha diversity of ´the protist community in summer (0.771) and in autumn (0.009) than the fungal community in the red soil (Fig. 5b, d), while the opposite results of stronger impacts of the fungal community on the protist community were observed in the black soil in summer and fluvo-aquic soil in autumn (Fig. 5a, c).

## Discussion

We here show that the application of nitrogen fertilizers affects the composition of the entire microbiome, particularly reducing soil protist diversity, with differences depending on plant growing season and soil type. We reveal that not only abiotic differences, but direct interactions within the microbiome drive the observed changes, with protists appearing as ecologically relevant microbiome components due to their predation manner on other microorganisms [30–32]. Protists were also most sensitive in the microbiome as their communities changed most strongly between fertilization treatments and seasons. Together with their small size, enormous diversity, high abundances, and broader presence in all environments [33], our results enforced the potential of protist communities as promising indicators that should be prioritized in future efforts to be implemented for, e.g., biomonitoring and pesticide indication. As protists were determined as key hubs in microbiome networks, changes in protist community might substantially influence soil microbiome associations through the top-control forces which need to be followed on.

Abiotic changes induced by fertilization were detected to structure microbiomes that were dependent on soil type and season. Numerous studies have demonstrated that long-term inorganic nitrogen fertilization changes soil bacterial and fungal communities through fertilization-induced changes in soil abiotics [9, 34, 35]. In this study, nitrogen fertilizer amendments significantly influenced the bacterial and fungal community composition but did not  influence their diversity, while the effect was much weaker compared with that on the protist community. The minor or non-significant change in pH (less than 0.42) and other abiotics under a relatively short-term fertilizer application period may partially explain the weak effect of nitrogen fertilization on bacterial and fungal community, as fertilization-induced abiotic changes might only emerge after years [36–38]. On the other side, protists might have a lower habitat niche breadths than bacteria [39], suggesting that protists are less tolerant than bacteria to environmental changes. All these results were in contrast to our first hypothesis. Previous studies revealed inconsistent conclusions on how nitrogen fertilization affects soil protist communities [23, 40]. We here reveal that short-term application of nitrogen fertilizers reduces protist diversity. In contrast to our hypothesis 2, straw amendments further lowered protist diversity, which could be attributed to the increased nitrogen retention under the straw incorporation in soil as demonstrated by ^15^N-urea tracing in our trial field in the black soil [16] and other studies [41, 42]. Based on the data, we cannot decipher the specific underlying mechanisms of protist diversity decreases, such as through direct effects or via changes in abiotic or biotic components. However, some competitively strong opportunistic taxa could be promoted that outcompete and therefore simplify protist communities [43]. As the loss of protist diversity induced by nitrogen fertilizers could subsequently trigger negative impacts on soil ecosystem stability and soil multifunctionality [2], it seems pivotal to better understand and manage soil microbiomes for a more sustainable agriculture. This integrative knowledge on microbiome functioning is, however, only achieved when the entire microbiome and not only individual parts are studied [36]. Therefore, investigations on potential soil microbiome interactions such as in this study are urgently needed.

In general, abiotics profoundly contributed to reductions in the alpha diversity of the soil protist community. As bacteria and fungi are a major prey for phagotrophic protists in soil [44–46], biotic interactions within the soil microbiome could also lead to reductions in protist diversity following fertilizations through bottom-up driving. However, bottom-up driving forces of bacteria and fungi on protists differed between soil types, which are explained by differences in moisture and C/N. The red soil site located in south China has much more rainfall than the other two sites in north China; thus, the red soil often contains higher moisture which makes it easy for protists to feed on their bacterial prey in water-filled soil pores [12]. Besides, fungi are more dominant in soil with higher C/N (like in the black and fluvo-aquic soils) than bacteria [13]. Therefore, the bacterial and fungal community differs in their effects on the protist diversity depending on soil abiotic properties.

Soil microbiome networks visualized the scenarios of biotic interactions and indicated that protist taxa are tightly linked within microbiomes as potential key microbiome controllers. Specific and different links between protist taxa and microbial taxa indicate that protist in these families feed in a taxon-specific manner on their potentially microbial prey. This confirms previous studies showing that protist taxa selectively feed on bacterial [30] and fungal prey [46]. Interestingly, however, we found variable links of some protist groups with different members of the microbiome in distinct soil types, seasons, and fertilization regimes. This suggests that these groups shift their predominant feeding and consequently the top-down impact on microbiome composition [45].

We found that the application of nitrogen fertilizers tightened soil microbiome association. This might be explained by changes of some taxa that were sensitive to nitrogen addition. The LEfSe analysis detected several nitrogen-susceptible taxa in the *Rhizobiales*, *Xanthomonadales*, and *Burkholderiales* which have previously been reported to be keystone taxa in agricultural ecosystems linked to rhizosphere function [47]. The application of nitrogen fertilizers reduced protist diversity and altered the links of key protist taxa in the microbiome networks, which also possibly contributed to the tightening of soil microbiome association. Alternatively, negative impacts of high nitrogen availability on soil microbiomes [13] could promote few strong associations with tightened microbiome complexity and consequently increase system stability to resist to adverse environmental conditions [48–50]. Lastly, the exogenous nutrient addition increased resource and food availability and subsequently could strengthen microbial interactions in order to enhance the efficiency of resource turnover that benefits plant growth [48, 49, 51]. Generally, the application of nitrogen fertilizers has tightened soil microbiome associations. Mechanisms that explain the observed differences in the microbiomes of different soil types yet need to be determined. Further results and discussions such as networks specific for sampling season are provided as supplementary information. Taken together, we propose that protists may be keystone of soil microbiome as protists strongly correlated and therefore potentially drive microbiome structure.

The study provides a vital scenario on the dynamics of how the soil microbiome responds to fertilization under a relatively short-term scale of two years and is the first to demonstrate that the application of nitrogen fertilizers is reducing protist community diversity and is tightening soil microbiome network associations in diverse agricultural soils. However, these findings are based on a 2-year field trial and need to be tested in other systems, with other crops, and on different continents. Besides, our results reveal that changes in abiotic parameters differed between soil types and varied across sampling season. This indicates that more repetitive samplings across broader temporal and spatial scales are needed to accurately depict abiotic parameters that determine soil microbiomes, as abiotic parameters might be changed further after long-term fertilization regime. Therefore, controlled experimental studies are needed to disentangle the main abiotic and biotic drivers of protist communities as well as that of entire soil microbiomes.

## Conclusions

Taken together, we show that the application of nitrogen fertilizers has a profound impact on soil microbiomes that differs between seasons and soil types, suggesting that studies focusing on a single soil type or sampling point cannot capture microbiome fluctuations that profoundly change microbiome composition throughout time. While fertilization also altered the bacterial and fungal community composition without affecting the diversity of these microbial groups, fertilization much more strongly reduced the diversity and changed the community composition of protists. Therefore, protist communities turned out as the most susceptible microbiome group to changes in soil conditions as induced by fertilization. Nevertheless, the changes in bacterial and fungal communities were found to be important bottom-up forces driving protist diversity. Several protist taxa were identified as potential keystone microbiome taxa, which might control microbiome functioning through top-down forces. This finding is of far-reaching importance to understand microbiome communities and consequently system’s stability. Future microbiome and soil biodiversity studies in general should include protist community analyses as otherwise important information on microbiome structures might be missing. Overall, we propose that a reduction of protist diversity induced by application of nitrogen fertilizers, especially if applied intensively, might have detrimental effects on agricultural soil ecosystem functioning and sustainability, especially over the long term.

## Methods

### Field site setup, management, and samplings

Three field experimental sites were located across a latitudinal gradient in China from north to central to south. The sites were chosen to represent the three main agricultural production areas in China (Additional file 1: Figure S7) and consisted of black soil in Gongzhuling (Jilin Province 43° 29′ N, 124° 47′ E, Mollisols), fluvo-aquic soil in Xuchang (Henan Province, 34° 2′ N, 113° 51′ E, Calcaric cambisol), and red soil in Taoyuan (Hunan Province, 28° 54′ N, 111° 29′ E, Ultisol). The average annual temperature in Gongzhuling (GZL), Xuchang (XC), and Taoyuan (TY) site was 6.1 °C, 14.4 °C, and 17.4 °C, respectively, and the wheat-maize rotation system was applied in Xuchang (XC) and Taoyuan (TY), while only maize was planted in Gongzhuling (GZL) due to the harsh winter. The geographical map (Additional file 1: Figure S7) was generated by the “*maps*,” “*maptools*,” “*grid*,” “*ggsn*,” “*legendMap*,” and “*ggplot*” packages in R platform. Three treatments, including no nitrogen fertilizer addition (control), nitrogen addition (N), and nitrogen plus straw addition (NS), were set up in triplicate plots (3.125 m × 8 m each plot) in each field site since 2015. All treatments were amended with the same dose of phosphorus (120 kg ha^−1^ P_2_O_5_) and potassium (120 kg ha^−1^ K_2_O) fertilizers. Nitrogen fertilizers were applied with an annual rate of 200 kg ha^−1^ nitrogen, and straw was applied with a rate of 5000 kg ha^−1^, consisting of dry maize straw from the last harvest that was cut into 2–3-cm lengths. All fertilizers and straw were used as a basal fertilizer before sowing and were hand applied in furrows and then covered as ridges.

Sampling was carried out at the maize heading stage in summer and before harvest in autumn after 2 years fertilization in 2016. Each sample was a composite of five cores randomly collected from the ridge. In each core, soil samples were collected from 5- to 10-cm depth on the ridge and between two plants, with top 5-cm soil layer removed to avoid exogenous disturbance (Additional file 1: Figure S7). In total, 54 soil samples were collected and immediately carried back to the laboratory on ice. Raw soil samples were sieved through 2-mm-diameter mesh. In addition, fine root and plant residue that can pass through the mesh was avoided before laboratory analysis.

### Soil physicochemical properties

Soil pH was determined in a 1:2.5 soil/water suspension with a glass electrode. Soil moisture was measured by the loss of weight after oven drying at 105 °C to constant weight. Total C (TC) and N (TN) were analyzed by an elemental analyzer (Vario EL III-Elementar, Germany). C/N was calculated by the ratio of TC and TN. Organic matter (OM) was measured using the K_2_Cr_2_O_7_-H_2_SO_4_ oxidation-reduction colorimetric method [52]. Dissolved organic carbon (DOC) was extracted by 0.5 M K_2_SO_4_ and measured by a TOC analyzer (Multi N/C 3100, Analytikjena, German). NO_3_^−^-N and NH_4_^+^-N were extracted with 1 M KCl and measured using a continuous flow analyzer (AA3, SEAL analytical, Germany).

### DNA extraction, PCR assays, and high-throughput sequencing

Soil subsamples for molecular analysis were kept in a freezer at − 80 °C before use. Total DNA was extracted from 0.4 g of soil using the PowerSoil DNA Isolation Kit (MO BIO laboratories, Carlsbad, USA). DNA quantity and quality were determined using a NanoDrop Spectrophotometer (NanoDrop Technologies Inc., Wilmington, DE, USA). Three commonly used primer sets were applied to metabarcoding approaches to study microbial communities targeting bacterial 16S rRNA genes [53], fungal [54] and protists [55] 18S rRNA genes, respectively (Additional file 1: Table S1). We acknowledge that the ITS region provides higher taxonomic resolution of fungal community [56], but we chose the 18S rRNA gene to allow phylogenetic tree construction [57] to acquire phylogenetic diversity of microbiomes [58]. PCR reactions were conducted in four parallels in 25 μl mixtures consisting of 12.5 μl Premix Taq™ (Takara Biotechnology, Dalian, China), 0.5 μl of each primer (10 μM), 2 μl of template which is five times diluted of extracted DNA, and 9.5 μl of sterilized ddH_2_O. Negative control samples were also included throughout the PCR assay to ensure reaction systems were not contaminated. PCR conditions for each primer set are detailed in Additional file 1: Table S1. PCR amplicons were extracted from 2% agarose gels and purified by using of the AxyPrep DNA Gel Extraction Kit (Axygen Biosciences, Union City, CA, USA) according to the manufacturer’s instructions and quantified through QuantiFluor™ -ST (Promega, USA). Purified amplicons were pooled in equimolar and sent for paired-end sequencing on an Illumina MiSeq PE 300 × 2 sequencer (Majorbio Bio-Pharm Technology Co. Ltd., Shanghai, China).

### Bioinformatics

Quantitative Insights into Microbial Ecology (QIIME) 1.90 [59] standard operation procedure was used to process raw sequences. In short, raw reads of individual samples were merged to paired-end reads (*multiple_join_paired_ends.py*), followed by removing barcodes from sequences (*multiple_extract_barcodes.py*) and demultiplex and quality filter sequence from data files (*multiple_split_libraries_fastq.py*). UPARSE was used for chimera removal, and operational taxonomic units (OTUs) were clustered at 97% sequence similarity and a representative sequence of each OTU was selected and used for taxonomic assignments [60]. Protist OTUs were taxonomically assigned by blasting against the Protist Ribosomal Reference (PR2) database (version_4.5) [61], while bacterial and fungal OTUs were assigned against the SILVA database (version_123) [62] at 90% minimum similarity (*assign_taxonomy.py*). Sequences obtained were rarefied at minimum number of sequences per sample (bacteria, 41,359; fungi, 37,002; eukaryotes, 36,242) for downstream analysis (*single_rarefaction.py*). *Rhodophyta*, *Streptophyta*, *Fungi*, *Opisthokonta_X*, *Metazoa*, and ambiguous taxa in *Eukaryotes* were excluded from the obtained protist OTU table (*filter_taxa_from_otu_table.py*). Representative sequences were aligned against the SILVA database [62] (*align_seqs.py*) to acquire the phylogenetic tree (*make_phylogeny.py*). Alpha diversity (faith_pd index) of bacterial, fungal, and protist communities were calculated based on the faith’s phylogenetic metric at OTU level (*alpha_diversity.py*). Nonmetric multidimensional scaling (NMDS) (*nmds.py*) was used to visualize dissimilarity of beta diversity based on the unweighted unifrac distance across different treatments according to the phylogenetic tree (*beta_diversity.py*) [63].

### Network analysis

In order to determine the effects of the application of nitrogen fertilizers on soil microbiome associations in the three types of soils, the underlying co-occurrences among protist, bacterial, and fungal taxa was depicted through network analysis using the CoNet plug-in in Cytoscape [64, 65]. The network analysis was performed at the family level of these three microbiomes to reduce the complexity of calculation and to ensure the accuracy of taxonomic information. Data filtering was performed prior to network construction to avoid zero values that could result in spurious correlations and to mitigate the seasonal variances, and therefore, the taxa represented in all samples were reserved [66]. To explore all the pairwise associations, correlation scores (Spearman correlation, Pearson correlation, Kullback-Leibler dissimilarity, Bray-Curtis dissimilarity and mutual information) were calculated [67, 68]. To avoid potential false-positive correlations and compositionality biases, the ReBoot procedure with 100 permutations was performed, and the resultant distribution was refined with 1000 bootstraps [66]. The *P* values of the five methods were integrated by the Brown method, and only significant correlations (*P* < 0.05) were retained for the downstream procedure [64]. The Benjamini-Hochberg multiple test was performed as a correlation after the Brown merging *P* values, which adjusted the false discovery rate, and the chance of false rejecting the null hypothesis is ≤ 0.05 [69]. The resulting correlations were imported into the Gephi platform and then visualized by the Frucherman Reingold algorithms [70]. The topology property parameters of the network, the degree, betweenness centrality, and closeness centrality of each node in the network were calculated by the plug-in Network Analyzer in Cytoscape [71]. The clustering coefficient and network density were chosen to reflect the changes of soil microbiomes associations as they are measurements of how close the nodes are embedded in their neighborhood and clustered together [72]. Degree, betweenness centrality (BC), and closeness centrality (CC) were selected to explore key hubs of networks [73]. The node with higher degree indicates it is highly connected with other nodes, and the node with higher BC value indicates that it is more closely connected to nodes in other modules of the network and is a candidate for a connector of the network. The node with higher CC value indicates it is more closely connected within the module of the network and is a candidate for a module hub of the network, and the node with higher both BC and CC values indicates it is the super-generalist of the network and a candidate for a hub of the network [74, 75]. Here, the cutoff value of the BC and CC of a node was set at 0.6 to explore the putative key hubs of each network. Furthermore, in order to investigate associations between protists and other microorganisms that can reflect the potential predatory-prey interactions, the edges linking protist to the bacterial or fungal taxa in at least two networks were calculated.

Network analysis was also used to determine the difference in soil microbiome associations between summer and autumn. Detailed descriptions are provided as supplementary information.

### Structural equation models

In order to quantify the importance of nitrogen addition and nitrogen plus straw addition on the changes of soil physicochemical properties and alpha diversity of bacterial, fungal, and protist communities in soils, SEMs were constructed. Based on biogeographical knowledge at present, the a priori and theoretical model assumed that (i) nitrogen fertilizers (N and NS treatments) directly influence alpha diversity of soil bacterial, fungal, and protist communities, respectively; (ii) nitrogen fertilizers indirectly affect alpha diversity of soil bacterial, fungal, and protist communities by changing soil physicochemical properties; and (iii) nitrogen fertilizers indirectly influence alpha diversity of soil bacterial, fungal, and protist communities by altering inter-kingdom interactions within soil microbiomes as a result of competition and predatory-prey interactions between the different microbial groups. Nitrogen addition (N) variables were created by assigning the value 1 to the nitrogen addition treatment and 0 to the control and nitrogen plus straw addition treatments. The nitrogen plus straw addition (NS) variables were created by assigning the value 1 to the nitrogen plus straw addition treatment and 0 to the control and nitrogen addition treatments. All of the measured soil physicochemical property indices were reduced in dimensions by nonmetric multidimensional scaling (NMDS), with the variances of soil properties being represented on the first axis of NMDS. Faith’s phylogenetic diversity metric was used to determine the alpha diversity of bacterial, fungal, and protist community at OUT level. All variables were standardized by *Z* transformation (mean = 0, standard deviation = 1) to improve normality using the *scale* function in R. The pairwise correlation among these variables was calculated by the Mantel test using the “*Ecodist*” package in R platform, and a covariance matrix of these variables was inserted into AMOS 17.0 (SPSS, Chicago, IL, USA) for SEM construction and analysis. Maximum likelihood estimation was used to fit the covariance matrix to the model [76]. Chi-square (*P* > 0.05), goodness-of-fit-index (GFI > 0.90), and root mean square error of approximation (RMSEA < 0.05) were measured to ensure the model adequately fit [77].

SEMs were also used to quantify effects of abiotic factors (nitrogen fertilizers, soil physicochemical properties) and biotic factors (bacterial community, fungal community) on the significant reductions (*P* < 0.05) in alpha diversity of protist community in the black soil in summer, fluvo-aquic soil in autumn, and red soil in summer and autumn. Detailed descriptions are provided as supplementary information.

### Statistics analysis

Statistical analyses were performed in R platform (3.3.1). One-way ANOVA was used to analyze differences in soil physicochemical properties, and alpha diversity among nitrogen fertilizer treatments and plant growth seasons (packages: *agricolae*, *car*), statistical differences were considered significant at *P* < 0.05. Duncan post hoc test was used to assess treatment differences in one-way ANOVA analyses. Variables were standardized by *Z* transformation (mean = 0, standard deviation = 1) to improve normality using *scale* function in R. Linear mixed models (LMM) were used to analyze the effects of nitrogen fertilization, soil type, season, and their interactive effects on alpha diversity of soil microbiome, with the plot position serial number (*i* _ *j*, where *i* and *j* are the row and column number of the plot, respectively) in the field as a random effect (packages: *lme4*, *lmerTest*) [78]. Permutation multivariate analysis of variance (PERMANOVA) was employed to assess the significance of the influential factors that differentiate soil physicochemical properties and microbiomes (packages: *vegan*, *adonis* function) [79]. The soil physicochemical properties data was input as column list of each variable (pH, moisture, etc.) for PERMANOVA followed by Bray-Curtis distance calculation, and the microbiomes data was input as matrix based on unweighted unifrac distance. Linear discriminant analysis effect size (LEfSe) was performed to investigate potential biomarkers (across five taxonomic levels, from phylum to genus for bacterial and fungal communities, from supergroup to family level for protist community) within soil microbiomes specifically enrich in one of the treatments in each soil type, in summer and autumn, respectively, based on *P* < 0.05 and a LDA score > 2.0 [80]. Biomarkers were color labeled on cladograms according to the PR2 taxonomy. Spearman correlations were calculated for depicting the relationship between the alpha diversity of the microbiomes and physicochemical properties in soils.

## Additional files

Additional file 1: **Figure S1.** Relative abundance of taxonomic composition of soil bacterial (A), fungal (B), and protist (C) community at phylum level, at class level, and at phylum level, respectively. **Figure S2.** LEfSe results revealed bacterial biomarkers (from phylum to genus level) sensitive to nitrogen fertilizers (no nitrogen addition (control) or nitrogen addition (N) or nitrogen + straw addition (NS)). **Figure S3.** LEfSe results revealed fungal biomarkers (from phylum level to genus level) sensitive to nitrogen fertilizers (no nitrogen addition (control) or nitrogen addition (N) or nitrogen + straw addition (NS)). **Figure S4.** Networks visualizing seasonal changes in co-occurrence patterns among protist, bacterial, and fungal taxa at family level across all soils in black soil, fluvo-aquic soil, and red soil, respectively. **Figure S5.** Beta-diversity of bacterial, fungal, and protist communities in each soil type under summer and autumn season, visualized by nonmetric multidimensional scaling (NMDS) based on unweighted unifrac phylogenetic distance metrics at OTU level. **Figure S6.** Putative key hubs in each network identified by betweenness centrality (BC) and closeness centrality (CC) of each node. **Figure S7.** Geographic location of three field experiment sites in China and diagram of fertilization and sampling arrangements. **Table S1.** Information of primers used in this study. **Table S2.** Physicochemical properties of the examined soil. **Table S3.** Bacterial biomarkers sensitive to nitrogen fertilizer treatments revealed by LEfSe analysis. **Table S4.** Fungal biomarkers sensitive to nitrogen fertilizer treatments revealed by LEfSe analysis. **Table S5.** Protist biomarkers sensitive to nitrogen fertilizer treatments revealed by LEfSe analysis. **Table S6.** Spearman correlations between alpha diversity of microbiomes and physicochemical properties in soils. **Table S7.** Node information of edges appearing in at least two networks linking protist to bacterial or fungal taxa in Fig. 3. **Table S8.** Topological indices used in this study for each network in Figure S4. **Table S9.** Nodes information of edges appearing in at least two networks linking protist to bacterial or fungal taxa in Figure S4. **Table S10.** The taxonomic information of protist nodes in Table 5. (DOCX 2031 kb)

Additional file 2: Details regarding network analyses to assess seasonal dynamics of soil microbiome associations and SEM to determine the importance abiotic factors and biotic factors on the significant reductions in the alpha diversity of the protist community. (DOCX 39 kb)
