# Supplementary material for: Protist communities are more sensitive to nitrogen fertilization than other microorganisms in diverse agricultural soils
Source: Microbiome. 2019 Feb 27;7:33. doi: 10.1186/s40168-019-0647-0 (PMC6393985; doi:10.1186/s40168-019-0647-0)
Supplement: Supplementary file 1 — Figure S1. Relative abundance of taxonomic composition of soil bacterial (A), fungal (B), and protist (C) community at phylum level, at class level, and at phylum level, respectively. Figure S2. LEfSe results revealed bacterial biomarkers (from phylum to genus level) sensitive to nitrogen fertilizers (no nitrogen addition (control) or nitrogen addition (N) or nitrogen + straw addition (NS)). Figure S3. LEfSe results revealed fungal biomarkers (from phylum level to genus level) sensitive to nitrogen fertilizers (no nitrogen addition (control) or nitrogen addition (N) or nitrogen + straw addition (NS)). Figure S4. Networks visualizing seasonal changes in co-occurrence patterns among protist, bacterial, and fungal taxa at family level across all soils in black soil, fluvo-aquic soil, and red soil, respectively. Figure S5. Beta-diversity of bacterial, fungal, and protist communities in each soil type under summer and autumn season, visualized by nonmetric multidimensional scaling (NMDS) based on unweighted unifrac phylogenetic distance metrics at OTU level. Figure S6. Putative key hubs in each network identified by betweenness centrality (BC) and closeness centrality (CC) of each node. Figure S7. Geographic location of three field experiment sites in China and diagram of fertilization and sampling arrangements. Table S1. Information of primers used in this study. Table S2. Physicochemical properties of the examined soil. Table S3. Bacterial biomarkers sensitive to nitrogen fertilizer treatments revealed by LEfSe analysis. Table S4. Fungal biomarkers sensitive to nitrogen fertilizer treatments revealed by LEfSe analysis. Table S5. Protist biomarkers sensitive to nitrogen fertilizer treatments revealed by LEfSe analysis. Table S6. Spearman correlations between alpha diversity of microbiomes and physicochemical properties in soils. Table S7. Node information of edges appearing in at least two networks linking protist to bacterial or fungal taxa in Fig. 3. Table S8. Topolog [file 40168_2019_647_MOESM1_ESM.docx]

**Additional file 1**

**Supplemental figures**

**Fig. S1 Relative abundance of taxonomic composition of soil bacterial (A), fungal (B) and protist (C) community at phylum level, at class level and at phylum level, respectively. Three fertilizer treatments including no nitrogen addition (control), nitrogen addition (N) and nitrogen +straw addition (NS), were applied to black soil, fluvo-aquic soil and red soil. Soil samplings were conducted in summer and autumn after two-year fertilizers application. In (B), class names were colour-coded on the right with respective phylum names listed above. In (C), phylum names were colour-coded on the right with respective supergroup names listed above. _X represents unidentified lower taxonomic ranks within the respective category.**


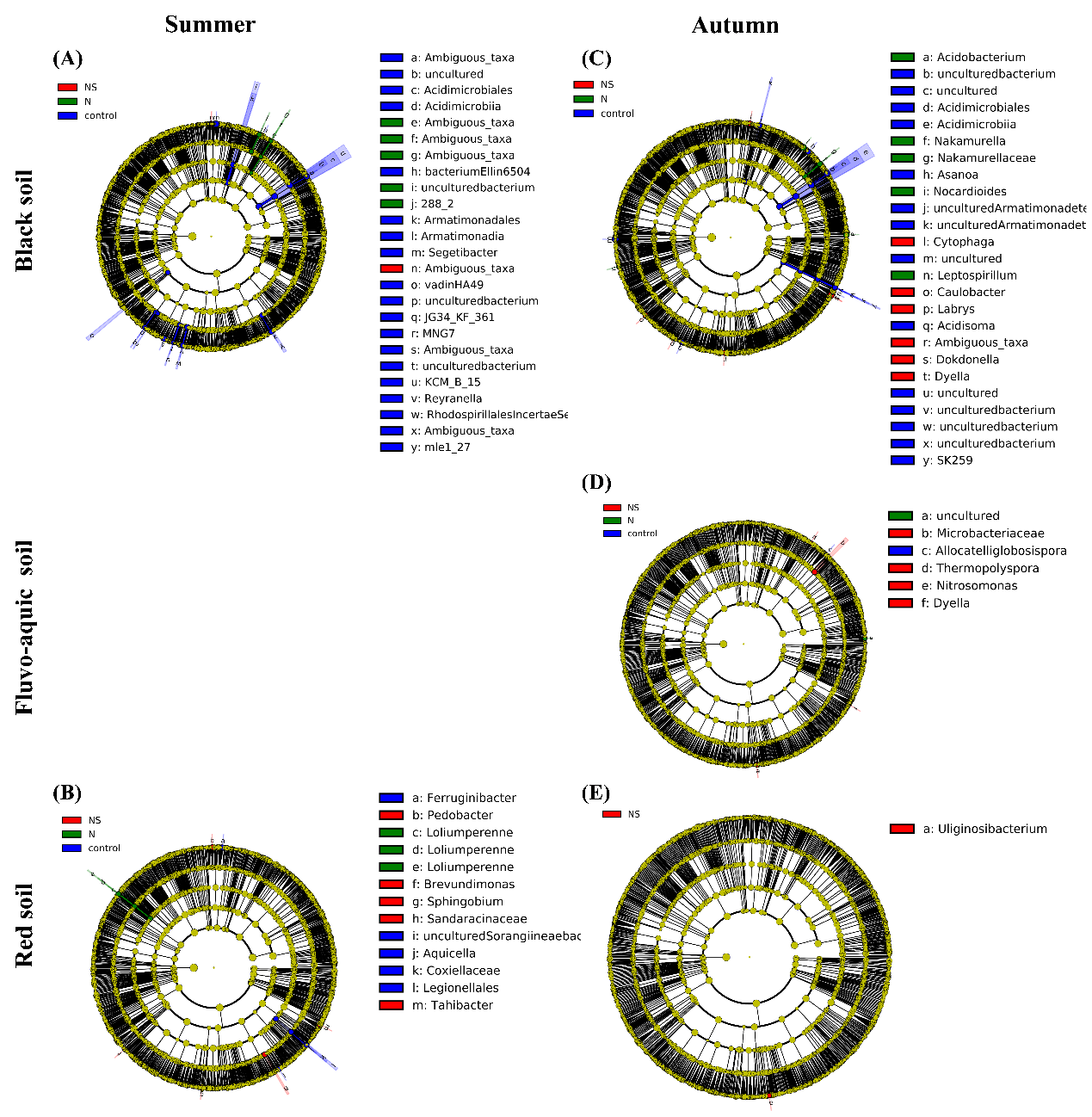


**Fig. S2 LEfSe results revealed bacterial biomarkers (from phylum to genus level) sensitive to nitrogen fertilizers (no nitrogen addition (control) or nitrogen addition (N) or nitrogen +straw addition (NS)). There are five circular rings in the cladogram, each circular ring deposit all taxa within a taxonomic level, the circular ring from inside to outside represents phylum, class, order, family, and genus, respectively. The node on the circular ring represents a taxon, affiliating within the taxonomic level. Taxa that had significantly higher relative abundance in a certain treatment within each soil type were color-coded within the cladogram according to the SILVA 123 taxonomy. Soil samplings were conducted in summer and autumn after two-year fertilizers application.**


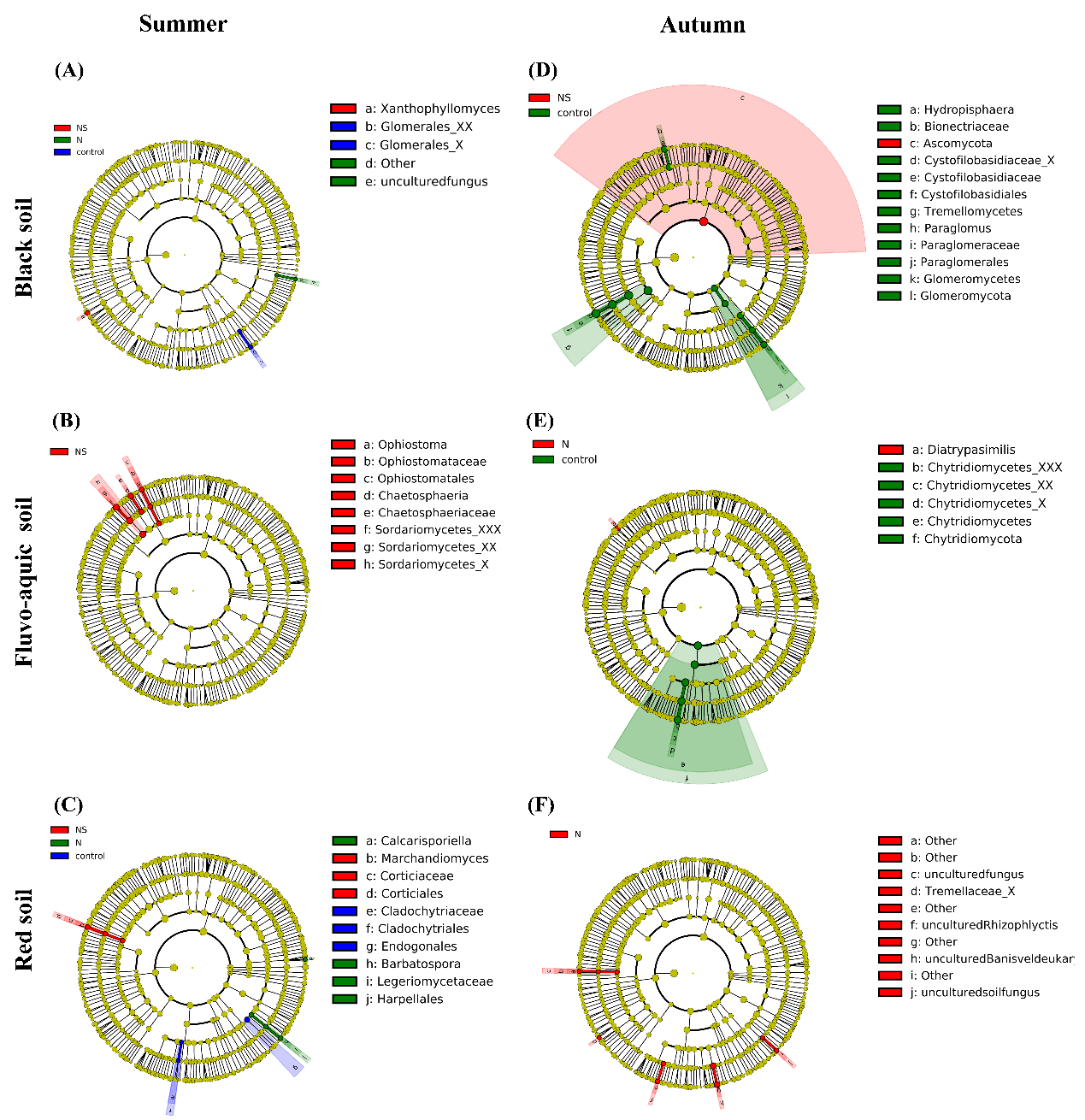


**Fig. S3 LEfSe results revealed fungal biomarkers (from phylum level to genus level) sensitive to nitrogen fertilizers (no nitrogen addition (control) or nitrogen addition (N) or nitrogen +straw addition (NS)). There are five circular rings in the cladogram, each circular ring deposit all taxa within a taxonomic level, the circular ring from inside to outside represents phylum, class, order, family, and genus, respectively. The node on the circular ring represents a taxon, affiliating within the taxonomic level. Taxa that had significantly higher relative abundance in a certain treatment within each soil type were color-coded within the cladogram according to the SILVA 123 taxonomy. Soil samplings were conducted in summer and autumn after two-year fertilizers applied. _X represents unidentified lower taxonomic ranks within the respective category.**

**
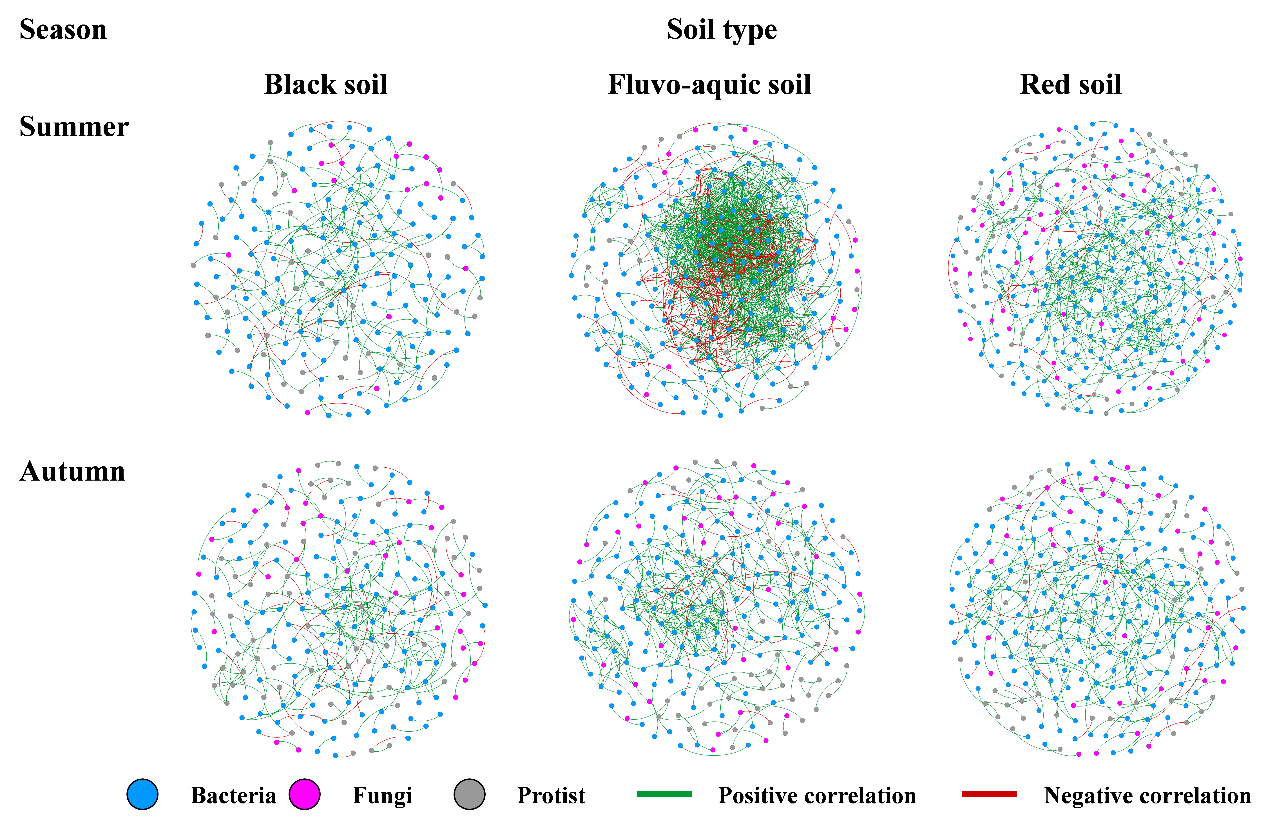
**

**Fig. S4 Networks visualizing seasonal changes in co-occurrence patterns among protist, bacterial and fungal taxa at family level across all soils in black soil, fluvo-aquic soil and red soil, respectively. Each network was constructed based on all fertilization treatments (including no nitrogen addition (control), nitrogen addition (N), nitrogen +straw addition (NS)) in a soil type in summer or autumn, respectively. Nodes filled in blue represent bacterial taxa, pink fungal taxa and grey protist taxa. Edges are colored according to interaction types, the positive correlation is labeled in green and the negative correlation was labeled with red.**

**
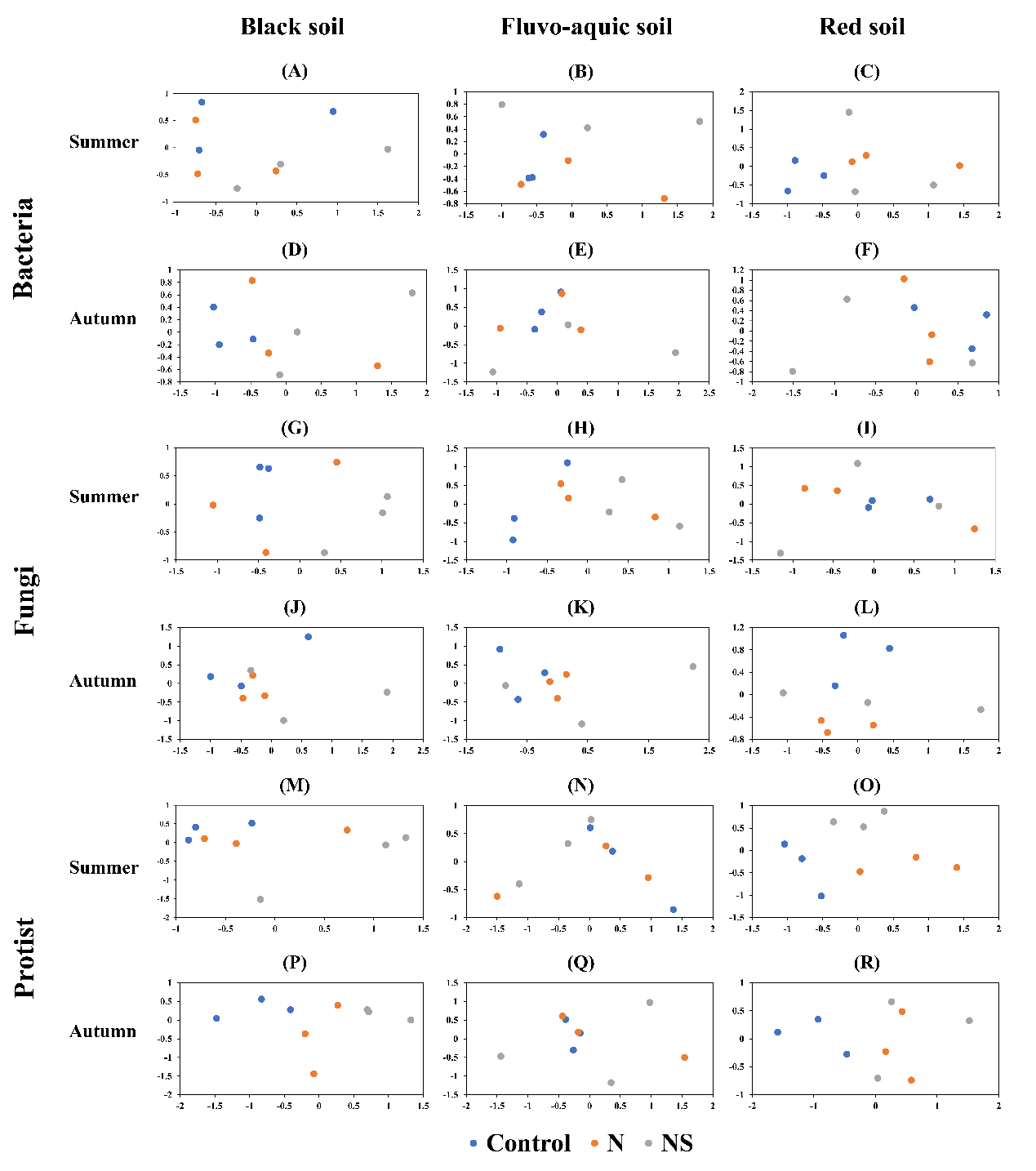
**

**Fig. S5 Beta-diversity of bacterial, fungal, and protist communities in each soil type under summer and autumn season, visualized by Nonmetric Multidimensional Scaling (NMDS) based on unweighted unifrac phylogenetic distance metrics at OTU level. The fertilization treatments includ no nitrogen addition (Control), nitrogen addition (N), and nitrogen plus straw addition (NS).**

**
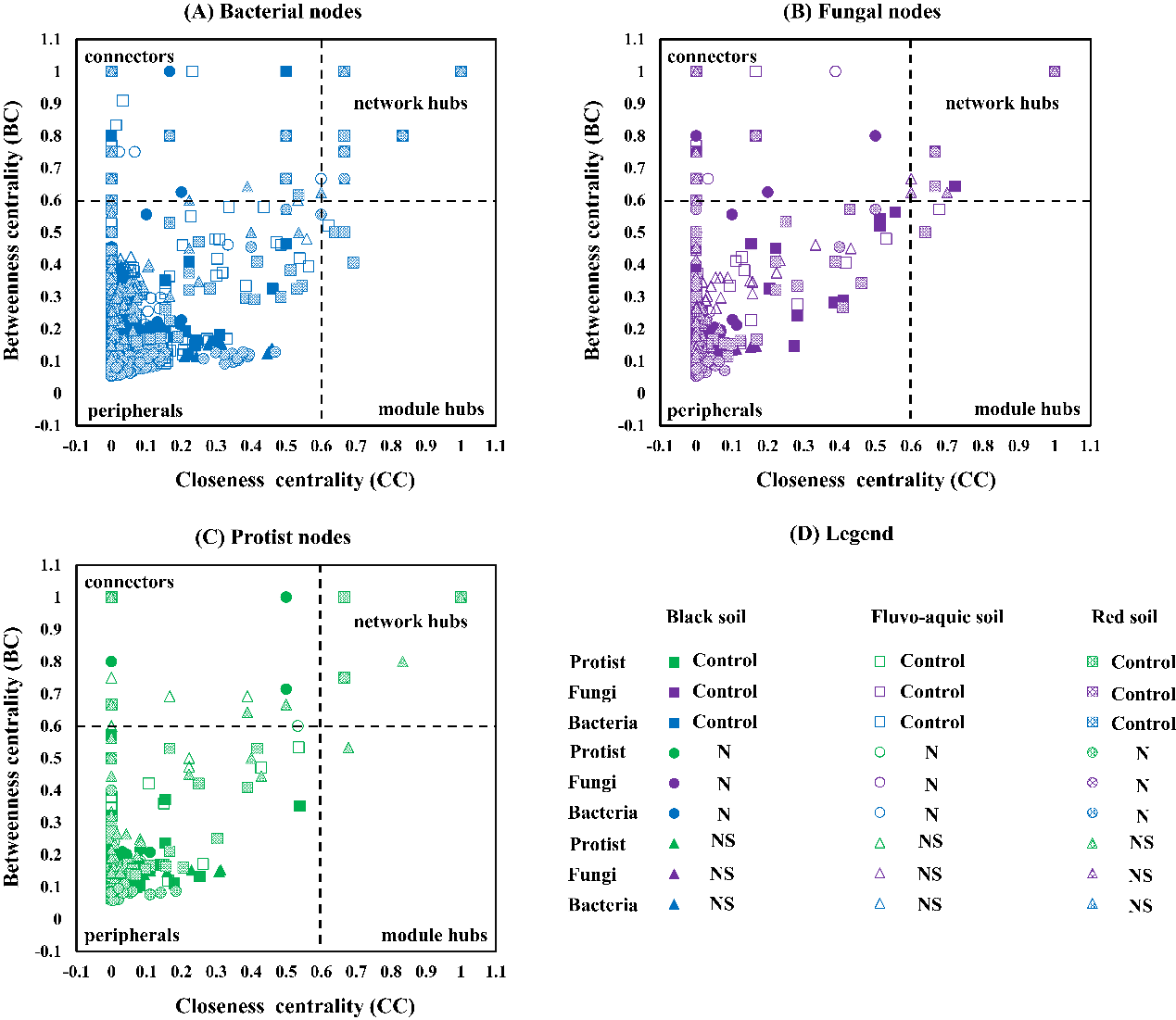
**

**Fig. S6 Putative key hubs in each network identified by Betweenness centrality (BC) and closeness centrality (CC) of each node. Each symbol represents a node in a network at family level. The shape of symbol denote fertilization treatments: rectangle (Control, no nitrogen addition), ellipse (N, nitrogen addition), triangle (NS, nitrogen plus straw addition). The colors of the symbols denote different taxonomic kingdoms: blue (bacteria), violet (fungi), green (protist). The fillings of the symbols denote different soil types: pure colour (black soil), hyaline (fluvo-aquic soil), shading (red soil). The cut-off value of the Betweenness centrality (BC) and closeness centrality (CC) was set at 0.6. Network hubs: BC > 0.6, CC > 0.6; connectors: BC > 0.6, CC < 0.6; module hubs: BC < 0.6, CC > 0.6, peripherals: BC < 0.6, CC < 0.6.**

**
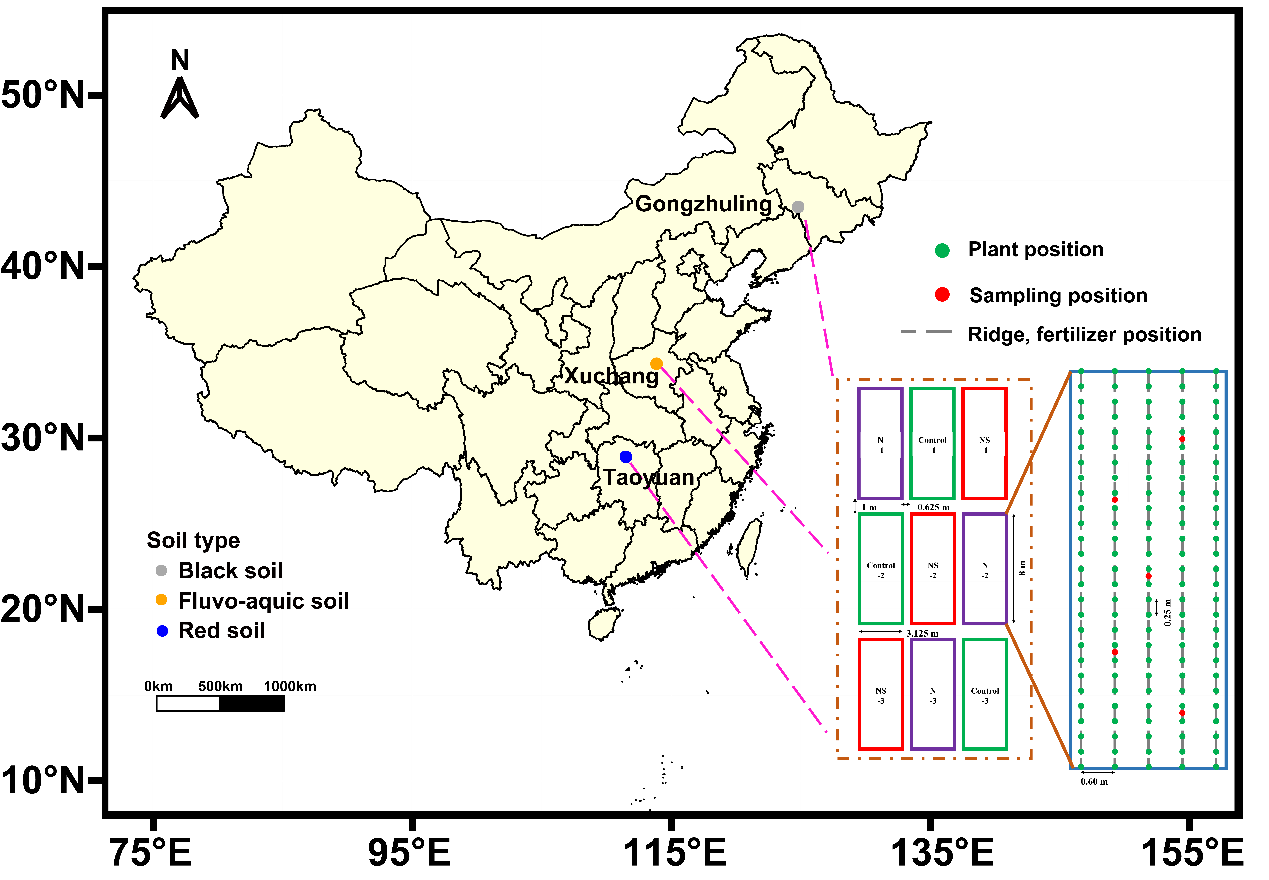
**

**Fig. S7 Geographic location of three field experiment sites in China and diagram of fertilization and sampling arrangements. Three sites located in Gongzhuling, Xuchang, and Taoyuan, corresponding to the black soil, the fluvo-aquic soil and the red soil, respectively. Three treatments, including no nitrogen fertilizer addition (control), nitrogen addition (N), nitrogen plus straw addition (NS), were set up in triplicate plots in each field site. Soils were collected between two plants on the ridge.**

**Supplemental tables**

**Table S1** Information of primers used in this study

|  | Primers | Primer sequence (5’- 3’) | Target gene | Target subfragment | Subfragment length (bp) | PCR reaction condition | References |
| --- | --- | --- | --- | --- | --- | --- | --- |
| Bacteria | FW-F515 | GTGCCAGC(A/C)GCCGCGGTAA | 16S | V4 | 291 | an initial denaturation at 95 °C for 3 minutes, followed by 27 cycles of 30 s at 95 °C, annealing for 30 s at 55 °C and elongation for 45 s at 72 °C, the last step being extension at 72 °C for 10 minutes. | [1] |
|  | REV-R806 | GGACTAC(G/A/C)(G/C)GGGTATCTAAT |  |  |  |  |  |
| Fungi | FW-F817 | TTAGCATGGAATAAT(A/G)(A/G)AATAGGA | 18S | V5-V7 | 379 | an initial denaturation at 95 °C for 3 minutes, followed by 35 cycles of 30 s at 95 °C, annealing for 30 s at 55 °C and elongation for 45 s at 72 °C, the last step being extension at 72 °C for 10 minutes | [2] |
|  | REV-R1196 | TCTGGACCTGGTGAGTTTCC |  |  |  |  |  |
| Protist | FW-TAReuk454FWD1 | CCA GCA (G/C)C(C/T) GCG GTA ATT CC | 18S | V4 | 418 | an initial denaturation at 95 °C for 5 minutes, followed by 10 cycles of 30 s at 94 °C, annealing for 45 s at 57 °C and elongation for 60 s at 72 °C, and followed by 25 cycles of 30 s at 94 °C, annealing for 45 s at 45 °C, 47 °C, 48 °C, 49 °C, respectively and elongation for 60 s at 72 °C, the last step being extension at 72 °C for 2 minutes. | [3] |
|  | REV-TAReukREV3 | ACT TTC GTT CTT GAT (C/T)(A/G)A |  |  |  |  |  |

**Table S2** Physicochemical properties of the examined soil and climate data of the sampling sites

| Season |  | pH | Moisture  % | C/N  ratio | OM  % | DOC  mg kg^-1^ | TC  % | TN  % | NH_4_^+^-N  mg kg^-1^ | NO_3_^—^N  mg kg^-1^ | | Temperature  Max/min,°C | Precipitation  mm |
| --- | --- | --- | --- | --- | --- | --- | --- | --- | --- | --- | --- | --- | --- |
| Summer | Black soil_control | 5.40±0.20 a1 | 13.94±1.23 a1 | 11.62±0.12 a1 | 1.99±0.02 | 94.69 ±8.69 | 1.76±0.02 | 0.152±0.003 | 5.57 ±1.67 | 17.24 ±11.40 a1 | | 32 / 19 | 0 |
|  | Black soil_ nitrogen addition | 5.16±0.09 ab1 | 11.81±0.23 b1 | 11.03±0.20 b1 | 2.01±0.03 | 92.11 ±8.80 | 1.72±0.04 | 0.156±0.003 | 4.59 ±2.79 | 57.10 ±25.36 b1 | |  |  |
|  | Black soil_ nitrogen +straw addition | 4.98±0.12 b1 | 13.01±0.14 ab1 | 11.15±0.08 b1 | 2.04±0.06 | 103.27 ±3.54 | 1.77±0.03 | 0.158±0.003 | 9.22 ±2.17 | 63.18 ±13.27 b1 | |  |  |
|  |  |  |  |  |  |  |  |  |  |  | | | |
|  | Fluvo-aquic soil_control | 7.79±0.11 | 8.63±1.91 | 12.74±0.22 | 1.64±0.17 | 64.68 ±9.54 | 1.69±0.12 | 0.133±0.008 | 2.19 ±0.37 a1 | 4.59 ±1.43 | | 30 / 23 | 88 |
|  | Fluvo-aquic soil_ nitrogen addition | 7.67±0.08 | 9.14±0.91 | 12.55±0.19 | 1.60±0.06 | 70.68 ±5.16 | 1.61±0.09 | 0.128±0.006 | 5.56 ±3.90 a1 | 50.33 ±6.92 | |  |  |
|  | Fluvo-aquic soil_ nitrogen +straw addition | 7.79±0.08 | 8.59±1.87 | 12.52±0.34 | 1.65±0.07 | 66.88 ±2.73 | 1.62±0.05 | 0.129±0.003 | 22.87 ±1.51 b1 | 129.68 ±111.40 | |  |  |
|  |  |  |  |  |  |  |  |  |  |  | | | |
|  | Red soil_control | 4.71±0.15 a2 | 23.29±0.90 | 9.12±0.10 | 2.26±0.05 | 186.89 ±19.92 | 1.73±0.08 | 0.189±0.007 | 7.42 ±1.61 | 16.90 ±1.72 a2 | | 32 / 24 | 291 |
|  | Red soil_ nitrogen addition | 4.34±0.09 b2 | 22.72±0.65 | 9.14±0.13 | 2.28±0.06 | 197.82 ±24.29 | 1.79±0.09 | 0.196±0.009 | 10.71 ±2.19 | 115.20 ±26.47 b2 | |  |  |
|  | Red soil_ nitrogen +straw addition | 4.40±0.14 b2 | 22.50±1.15 | 8.96±0.29 | 2.25±0.39 | 187.10 ±28.38 | 1.70±0.17 | 0.189±0.013 | 7.57 ±1.84 | 162.71 ±15.19 c2 | |  |  |
|  |  |  |  |  |  |  |  |  |  |  | | | |
| Autumn | Black soil_control | 5.28±0.07 a3 | 22.11±0.24 | 12.26±0.18 | 2.38±0.09 | 78.87±3.76 c1 | 1.75±0.1 | 0.143±0.006 b1 | 2.76±0.57 b2 | 3.56±1.65 | 23 / 11 | | 0 |
|  | Black soil_ nitrogen addition | 5.06±0.11 b3 | 22.45±1.61 | 12.54±0.68 | 2.35±0.17 | 87.13±2.71 b1 | 1.79±0.1 | 0.143±0.002 b1 | 7.37±2.61 a2 | 9.22±0.84 |  |  |  |
|  | Black soil_ nitrogen +straw addition | 4.97±0.12 b3 | 22.66±0.33 | 11.92±0.36 | 2.46±0.35 | 99.28±5.31 a1 | 1.86±0.06 | 0.156±0.003 a1 | 7.58±0.26 a2 | 11.67±7.91 |  |  |  |
|  |  |  |  |  |  |  |  |  |  |  | | | |
|  | Fluvo-aquic soil_control | 7.77±0.06 ab4 | 12.9±2.76 | 14.39±0.26 | 1.68±0.13 b1 | 58.11±7.17 | 1.69±0.14 | 0.117±0.007 | 2.72±0.64 | 6.97±2.23 | 25 / 19 | | 4 |
|  | Fluvo-aquic soil_ nitrogen addition | 7.66±0.07 b4 | 12.1±0.39 | 14.03±0.38 | 2.07±0.27 a1 | 61.6±6.09 | 1.69±0.09 | 0.120±0.003 | 3.61±0.23 | 21.46±9 |  |  |  |
|  | Fluvo-aquic soil_ nitrogen +straw addition | 7.86±0.11 a4 | 11.15±0.76 | 13.9±0.21 | 1.87±0.11 ab1 | 62.92±6.76 | 1.69±0.01 | 0.121±0.003 | 6.22±4.18 | 13.28±10.83 |  |  |  |
|  |  |  |  |  |  |  |  |  |  |  | | | |
|  | Red soil_control | 4.98±0.06 a5 | 21.3±0.61 | 8.94±0.13 | 2.8±0.3 | 173.45±8.64 | 1.76±0.08 | 0.197±0.007 | BDL b3 | 3.82±0.18 b3 | 30 / 21 | | 0 |
|  | Red soil_ nitrogen addition | 4.88±0.07 a5 | 20.87±1.31 | 8.98±0.29 | 2.82±0.31 | 173.2±20.78 | 1.73±0.17 | 0.192±0.015 | BDL b3 | 3.63±0.41 b3 |  |  |  |
|  | Red soil_ nitrogen +straw addition | 4.66±0.05 b5 | 21.49±1.6 | 9.19±0.18 | 2.73±0.17 | 168.74±14.86 | 1.77±0.14 | 0.193±0.016 | 3.16±1.13 a3 | 5.65±0.50 a3 |  |  |  |

Abbreviation: OM: organic matter; TC: total content of carbon; TN: total content of nitrogen; C/N: the ratio of TC and TN; Moisture: soil water content; DOC: dissolve organic carbon, BDL: Below detection limit; NH_4_^+^-N: ammonium nitrogen; NO_3_^-^-N: nitrate nitrogen.

One-way ANOVA was used to test difference significance among treatments, only significant differences between treatments was labeled with letter and digit, and the digit behind the letter meant different comparison groups. Duncan post-hoc test was used in One-way ANOVA. Temperature and precipitation were represented by the average and the sum value of the past three days before the sampling date, respectively, and the data was collected from National Meteorological Information Center of China (data.cma.cn)

Three fertilization treatments include no nitrogen addition (control), nitrogen addition, nitrogen +straw addition, soil samplings were conducted in summer and autumn after two-year fertilization.

**Table S3** Bacterial biomarkers sensitive to nitrogen fertilizer treatments revealed by LEfSe analysis

| Season | Soil type | Phylum | Class | Order | Family | Genus | Enriched in the treatment |
| --- | --- | --- | --- | --- | --- | --- | --- |
| Summer | Black soil | Actinobacteria | Acidimicrobiia |  |  |  | control |
|  |  | Actinobacteria | Acidimicrobiia | Acidimicrobiales |  |  | control |
|  |  | Actinobacteria | Acidimicrobiia | Acidimicrobiales | uncultured |  | control |
|  |  | Actinobacteria | AcidimicrobiIa | Aaidimicrobiales | uncultured | Ambiguous_taxa | control |
|  |  | Actinobacteria | MB_A2_108 | Ambiguous_taxa |  |  | N |
|  |  | Actinobacteria | MB_A2_108 | Ambiguous_taxa | Ambiguous_taxa |  | N |
|  |  | Actinobacteria | MB_A2_108 | Ambiguous_taxa | Ambiguous_taxa | Ambiguous_taxa | N |
|  |  | Actinobacteria | Thermoleophilia | Gaiellales | uncultured | bacteriumEllin6504 | control |
|  |  | Actinobacteria | Thermoleophilia | Solirubrobacterales | 288_2 |  | N |
|  |  | Actinobacteria | Thermoleophilia | Solirubrobacterales | 288_2 | unculturedbacterium | N |
|  |  | Armatimonadetes | Armatimonadia |  |  |  | control |
|  |  | Armatimonadetes | Armatimonadia | Armatimonadales |  |  | control |
|  |  | Bacteroidetes | Sphingobacteriia | Sphingobacteriales | Chitinophagaceae | Segetibacter | control |
| Summer | Black soil | Bacteroidetes | Sphingobacteriia | Sphingobacteriales | Sphingobacteriaceae | Ambiguous_taxa | NS |
|  |  | Planctomycetes | vadinHA49 |  |  |  | control |
|  |  | Proteobacteria | Alphaproteobacteria | Rhizobiales | JG34_KF_361 |  | control |
|  |  | Proteobacteria | Alphaproteobacteria | Rhizobiales | JG34_KF_ 361 | unculturedbacterium | control |
|  |  | Proteobacteria | Alphaproteobacteria | Rhizobiales | MNG7 |  | control |
|  |  | Proteobacteria | Alphaproteobacteria | Rhodospirillales | Acetobacteraceae | Ambiguous_taxa | control |
|  |  | Proteobacteria | Alphaproteobacteria | Rhodospirillales | KCM_B_15 |  | control |
|  |  | Proteobacteria | Alphaproteobacteria | Rhodospirillales | KCM_B_15 | unculturedbacterium | control |
|  |  | Proteobacteria | Alphaproteobacteria | Rhodospirillales | RhodospirillalesIncertaeSedis |  | control |
|  |  | Proteobacteria | Alphaproteobacteria | Rhodospinillales | RhodospirillalesIncertaeSedis | Reyranella | control |
|  |  | Proteobacteria | Deltaproteobacteria | Myxococcales | mle1_27 |  | control |
|  |  | Proteobacteria | Deltaproteobacteria | Myxococcales | mle1_27 | Ambiguous_taxa | control |
|  | Red soil | Bacteroidetes | Sphingobacteria | Sphingobacteriales | Chitinophagaceae | Ferruginibacter | control |
|  |  | Bacteroidetes | Sphingobacteriia | Sphingobacteriales | Sphingobacteriaceae | Pedobacter | NS |
| Summer | Red soil | Cyanobacteria | Chloroplast | Loliumperenne |  |  | N |
|  |  | Cyanobacteria | Chloroplast | Loliumperenne | Loliumperenne |  | N |
|  |  | Cyanobacteria | Chloroplast | Loliumperenne | Loliumperenne | Loliumperenne | N |
|  |  | Proteobacteria | Alphaproteobacteria | Caulobacterales | Caulobacteraceae | Brevundimonas | NS |
|  |  | Proteobacteria | Alphaproteobacteria | Sphingomonadales | Sphingomonadaceae | Sphingobium | NS |
|  |  | Proteobacteria | Deltaproteobacteria | Myxococcales | mle1_27 | unculturedSorangiineaebacterium | control |
|  |  | Proteobacteria | Deltaproteobacteria | Myxococcales | Sandaracinaceae |  | NS |
|  |  | Proteobactena | Gammaproteobacteria | Legionellales |  |  | control |
|  |  | Proteobacteria | Gammaproteobacteria | Legionellales | Coxiellaceae |  | control |
|  |  | Proteobacteria | Gammaproteobacteria | Legionellales | Coxiellaceae | Aquicella | control |
|  |  | Proteobacteria | Gammaproteobacteria | Xanthomonadales | Xanthomonadaceae | Tahibacter | NS |
| Autumn | Black soil | Acidobacteria | Acidobacteria | Acidobacteriales | Acidobacteriaceae_Subgroup1 | Acidobacterium | N |
|  |  | Actinobacteria | Acidimicrobiia |  |  |  | control |
|  |  | Actinobacteria | Acidimicrobiia | Acidimicrobiales |  |  | control |
| Autumn | Black soil | Actinobacteria | Acidimicrobiia | Acidimicrobiales | uncultured |  | control |
|  |  |  |  |  |  |  |  |
|  |  | Actinobacteria | Acidimicrobiia | Acidimicrobiales | uncultured | unculturedbacterium | control |
|  |  | Actinobacteria | Actinobacteria | Frankiales | Nakamurellaceae |  | N |
|  |  | Actinobacteria | Actinobacteria | Frankiales | Nakamurellaceae | Nakamurella | N |
|  |  | Actinobacteria | Actinobacteria | Micromonosporales | Micromonosporaceae | Asanoa | control |
|  |  | Actinobacteria | Actinobacteria | Propionibacteriales | Nocardioidaceae | Nocardioides | N |
|  |  | Armatimonadetes | Armatimonadetesbacterium |  |  |  | control |
|  |  | Armatimonadetes | Armatimonadetesbacterium | unculturedArmatimonadetesbacterium | unculturedArmatimonadetesbacterium |  | control |
|  |  | Bacteroidetes | Cytophagia | Cytophagales | Cytophagaceae | Cytophaga | NS |
|  |  | Gemmatimonadetes | Gemmatimonadetes | Gemmatimonadales | Gemmatimonadaceae | uncultured | control |
|  |  | Nitrospirae | Nitrospira | Nitrospirales | Nitrospiraceae | Leptospirillum | N |
|  |  | Proteobacteria | Alphaproteobacteria | Caulobacterales | Caulobacteraceae | Caulobacter | NS |
|  |  | Proteobacteria | Alphaproteobacteria | Rhizobiales | Xanthobacteraceae | Labrys | NS |
| Autumn | Black soil | Proteobacteria | Alphaproteobacteria | Rhodospirillales | Acetobacteraceae | Acidisoma | control |
|  |  | Proteobacteria | Betaproteobacteria | Burkholderiales | Alcaligenaceae | Ambiguous_taxa | NS |
|  |  | Proteobacteria | Gammaproteobacteria | Xanthomonadales | Xanthomonadaceae | Dokdonella | NS |
|  |  | Proteobacteria | Gammaproteobacteria | Xanthomonadales | Xanthomonadaceae | Dyella | NS |
|  |  | Proteobacteria | Gammaproteobacteria | Xanthomonadales | Xanthomonadaceae | uncultured | control |
|  |  | Proteobacteria | SK259 |  |  |  | control |
|  |  | Proteobacteria | SK259 | unculturedbacterium |  |  | control |
|  |  | Proteobacteria | SK259 | unculturedbacterium | unculturedbacterium |  | control |
|  |  | Proteobacteria | SK259 | unculturedbacterium | unculturedbacterium | unculturedbacterium | control |
|  | Fluvo-aquic soil | Acidobacteria | Acidobacteria | Acidobacteriales | Acidobacteriaceae_ Subgroupl | uncultured | N |
|  |  | Actinobacteria | Actinobacteria | Micrococcales | Microbacteriaceae |  | NS |
|  |  | Actinobacteria | Actinobacteria | Micromonosporales | Micromonosporaceae | Allocatelliglobosispora | control |
|  |  | Actinobacteria | Actinobacteria | Streptosporangiales | Streptosporangiaceae | Thermopolyspora | NS |
|  |  | Proteobacteria | Betaproteobacteria | Nitrosomonadales | Nitrosomonadaceae | Nitrosomonas | NS |
| Autumn | Fluvo-aquic soil | Proteobacteria | Gammaproteobacteria | Xanthomonadales | Xanthomonadaceae | Dyella | NS |
|  | Red soil | Proteobacteria | Betaproteobacteria | Rhodocyclales | Rhodocyclaceae | Uliginosibacterium | NS |

**Table S4** Fungal biomarkers sensitive to nitrogen fertilizer treatments revealed by LEfSe analysis

| Season | Soil type | Phylum | Class | Order | Family | Genus | Enriched in the treatment |
| --- | --- | --- | --- | --- | --- | --- | --- |
| Summer | Black soil | Basidiomycota | Tremellomycetes | Cystofilobasidiales | Cystofilobasidiaceae | Xanthophyllomyces | NS |
|  |  | Glomeromycota | Glomeromycetes | Glomerales | Glomerales_X | | control |
|  |  | Glomeromycota | Glomeromycetes | Glomerales | Glomerales_X | Glomerales_XX | control |
|  |  | Zygomycota | Zygomycota_X | Zoopagales | unculturedfungus | | N |
|  |  | Zygomycota | Zygomycota_X | Zoopagales | unculturedfungus | Other | N |
|  | Fluvo-aquic soil | Ascomycota | Sordariomycetes | Ophiostomatales | |  | NS |
|  |  | Ascomycota | Sordariomycetes | Ophiostomatales | Ophiostomataceae | | NS |
|  |  | Ascomycota | Sordariomycetes | Ophiostomatales | Ophiostomataceae | Ophiostoma | NS |
|  |  | Ascomycota | Sordariomycetes | Sordariales | Chaetosphaeriaceae | | NS |
|  |  | Ascomycota | Sordariomycetes | Sordariales | Chaetosphaeriaceae | Chaetosphaeria | NS |
|  |  | Ascomycota | Sordariomycetes | Sordariomycetes_X | |  | NS |
|  |  | Ascomycota | Sordariomycetes | Sordariomycetes_X | Sordariomycetes_XX | | NS |
|  |  | Ascomycota | Sordariomycetes | Sordariomycetes_X | Sordariomycetes_XX | Sordariomycetes_XXX | NS |
| Summer | Red soil | Ascomycota | Ascomycota_X | Ascomycota_XX | Ascomycota_XXX | Calcarisporiella | N |
|  |  | Basidiomycota | Agaricomycetes | Corticiales | |  | NS |
|  |  | Basidiomycota | Agaricomycetes | Corticiales | Corticiaceae | | NS |
|  |  | Basidiomycota | Agaricomycetes | Corticiales | Corticiaceae | Marchandiomyces | NS |
|  |  | Chytridiomycota | Chytridiomycetes | Cladochytriales | |  | control |
|  |  | Chytridiomycota | Chytridiomycetes | Cladochytriales | Cladochytriaceae | | control |
|  |  | Zygomycota | Zygomycota_X | Endogonales | |  | control |
|  |  | Zygomycota | Zygomycota_X | Harpellales | |  | N |
|  |  | Zygomycota | Zygomycota_X | Harpellales | Legeriomycetaceae | | N |
|  |  | Zygomycota | Zygomycota_X | Harpellales | Legeriomycetaceae | Barbatospora | N |
| Autumn | Black soil | Ascomycota | |  |  |  | NS |
|  |  | Ascomycota | Sordariomycetes | Hypocreales | Bionectriaceae | | control |
|  |  | Ascomycota | Sordariomycetes | Hypocreales | Bionectriaceae | Hydropisphaera | control |
|  |  | Basidiomycota | Tremellomycetes | |  |  | control |
| Autumn | Black soil | Basidiomycota | Tremellomycetes | Cystofilobasidiales | | | control |
|  |  | Basidiomycota | Tremellomycetes | Cystoflobasidiales | Cystofilobasidiaceae | | control |
|  |  | Basidiomycota | Tremellomycetes | Cystoflobasidiales | Cystofilobasidiaceae | Cystofilobasidiaceae_X | control |
|  |  | Glomeromycota | |  |  |  | control |
|  |  | Glomeromycota | Glomeromycetes | |  |  | control |
|  |  | Glomeromycota | Glomeromycetes | Paraglomerales | |  | control |
|  |  | Glomeromycota | Glomeromycetes | Paraglomerales | Paraglomeraceae | | control |
|  |  | Glomeromycota | Glomeromycetes | Paraglomerales | Paraglomeraceae | Paraglomus | control |
|  | Fluvo-aquic soil | Ascomycota | Sordariomycetes | Xylariales | Xylariales_X | Diatrypasimilis | N |
|  |  | Chytridiomycota | |  |  |  | control |
|  |  | Chytridiomycota | Chytridiomycetes | |  |  | control |
|  |  | Chytridiomycota | Chytridiomycetes | Chytridiomycetes_X | | | control |
|  |  | Chytridiomycota | Chytridiomycetes | Chytridiomycetes_X | Chytridiomycetes_XX | | control |
|  |  | Chytridiomycota | Chytridiomycetes | Chytridiomycetes_X | Chytridiomycetes_XX | Chytridiomycetes_XXX | control |
| Autumn | Red soil | Basidiomycota | Agaricomycetes | unculturedfungus | |  | N |
|  |  | Basidiomycota | Agaricomycetes | unculturedfungus | Other |  | N |
|  |  | Basidiomycota | Agaricomycetes | unculturedfungus | Other | Other | N |
|  |  | Basidiomycota | Tremellomycetes | Tremellales | Tremellaceae | Tremellaceae_X | N |
|  |  | Chytridiomycota | Chytridiomycetes | Chytridiales | Uncultured Rhizophlyctis | | N |
|  |  | Chytridiomycota | Chytridiomycetes | Chytridiales | Uncultured Rhizophlyctis | Other | N |
|  |  | Chytridiomycota | Chytridiomycetes | Spizellomycetales | Uncultured Banisveldeukaryote | | N |
|  |  | Chytridiomycota | Chytridiomycetes | Spizellomycetales | Uncultured Banisveldeukaryote | Other | N |
|  |  | Zygomycota | Zygomycota_X | Endogonales | Uncultured soil fungus | | N |
|  |  | Zygomycota | Zygomycota_X | Endogonales | Uncultured soil fungus | Other | N |

**Table S5** Protist biomarkers sensitive to nitrogen fertilizer treatments revealed by LEfSe analysis

| Season | Soil type | Supergroup | Phylum | Class | Order | Family | Enriched in the treatment |
| --- | --- | --- | --- | --- | --- | --- | --- |
| Summer | Black soil | Amoebozoa | Conosa | Variosea | Variosea_X | Phalansteriidae | control |
|  |  | Archaeplastida | Chlorophyta | Chlorophyceae | Sphaeropleales |  | N |
|  |  | Archaeplastida | Chlorophyta | Chlorophyceae | Sphaeropleales | Sphaeropleales_ X | N |
|  |  | Archaeplastida | Chlorophyta | Mamiellophyceae |  |  | control |
|  |  | Archaeplastida | Chlorophyta | Mamiellophyceae | Dolichomastigales |  | control |
|  |  | Archaeplastida | Chlorophyta | Mamiellophyceae | Dolichomastigales |  | control |
|  |  | Rhizaria | Cercozoa | Filosa_ Imbricatea | Euglyphida |  | N |
|  |  | Rhizaria | Cercozoa | Filosa_ Imbricatea | Euglyphida | Euglyphida_X | N |
|  |  | Rhizaria | Cercozoa | Filosa_ Imbricatea | Euglyphida | Trinematidae | N |
|  |  | Rhizaria | Cercozoa | Filosa_ Sarcomonadea | Glissomonadida | Glissomonadida_ X | NS |
|  |  | Stramenopiles | Ochrophyta | Chrysophyceae | Chrysophyceae_X | Chrysophyceae_ Clade_F | N |
|  |  | Stramenopiles | Stramenopiles_ X | MAST | MAST_3 |  | control |
|  |  | Stramenopiles | Stramenopiles_ X | MAST | MAST_7 |  | control |
| Summer | Black soil | Stramenopiles | Stramenopiles_ X | MAST | MAST_7 | MAST_7A | control |
|  |  | Stramenopiles | Stramenopiles_ X | Oomycota | Oomycota_X | Oomycota_ XX | control |
|  | Red soil | Alveolata | Ciliophora | Oligohymenophorea | Scuticociliatia |  | NS |
|  |  | Alveolata | Ciliophora | Oligohymenophorea | Scuticociliatia | Scuticociliatia_X | NS |
|  |  | Alveolata | Dinophyta |  |  |  | control |
|  |  | Amoebozoa | Conosa |  |  |  | control |
|  |  | Amoebozoa | Conosa | Variosea |  |  | control |
|  |  | Amoebozoa | Conosa | Variosea | Variosea_X |  | control |
|  |  | Hacrobia |  |  |  |  | control |
|  |  | Hacrobia | Centroheliozoa | Centroheliozoa X | Pterocystida | Pterocystida_X | control |
|  |  | Opisthokonta | Choanoflagellida | Choanoflagellatea | Craspedida | Salpingoecidae_ Group_C1 | NS |
|  |  | Rhizaria | Cercozoa | Endomyxa | Vampyrellida | Vampyrellidae | control |
|  |  | Rhizaria | Cercozoa | Filosa_Sarcomonadea | Glissomonadida | Allapsidae | N |
|  |  | Rhizaria | Cercozoa | Filosa_Sarcomonadea | Glissomonadida | Dujardinidae | NS |
| Autumn | Black soil | Amoebozoa | Conosa | Mycetozoa_Myxogastrea |  |  | control |
|  |  | Amoebozoa | Conosa | Variosea | ATCC50593_FLamella_WIM80 lineage | WIM80 lineage | control |
|  |  | Amoebozoa | Conosa | Variosea | Variosea_X | Variosea XX | control |
|  |  | Hacrobia | Centroheliozoa |  |  |  | control |
|  |  | Hacrobia | Centroheliozoa | Centroheliozoa_X |  |  | control |
|  |  | Hacrobia | Telonemia | Telonemia_X | Telonemia_XX | Telonemia_Group_2 | control |
|  |  | Opisthokonta | Choanoflagellida | Choanoflagellatea | Craspedida | Monosigidae_Group_O | control |
|  |  | Rhizaria | Cercozoa | Endomyxa |  |  | control |
|  |  | Rhizaria | Cercozoa | Endomyxa | Vampyrellida |  | control |
|  |  | Rhizaria | Cercozoa | Endomyxa | Vampyrellida | Leptophryidae | control |
|  |  | Rhizaria | Cercozoa | Endomyxa | Vampyrelida | sm27_lineage | control |
|  |  | Rhizaria | Cercozoa | Endomyxa | Vampyrellida | Vampyrellidae | N |
|  |  | Stramenopiles | Stramenopiles_X | MAST | MAST_3 |  | control |
|  |  | Stramenopiles | Stramenopiles_X | MAST | MAST_7 | MAST_7A | control |
| Autumn | Fluvo-aquic soil | Amoebozoa | Conosa | Variosea | Variosea_X | WIM_1_lineage | control |
|  | Red soil | Alveolata | Ciliophora | Litostomatea | Haptoria | Enchelyidae | N |
|  |  | Amoebozoa | Conosa | Variosea | Variosea_X | Acramoebidae | control |
|  |  | Amoebozoa | Conosa | Variosea | Variosea_X | AND16_lineage | control |
|  |  | Amoebozoa | Conosa | Variosea | Variosea_X | Phalansteriidae | control |
|  |  | Amoebozoa | Lobosa | Discosea_Longamoebia |  |  | control |
|  |  | Amoebozoa | Lobosa | Discosea_Longamoebia | Thecamoebida |  | control |
|  |  | Amoebozoa | Lobosa | Discosea_Longamoebia | Thecamoebida | Thecamoebidae | control |
|  |  | Archaeplastida | Chlorophyta | Trebouxiophyceae | Trebouxiophyceae_X |  | control |
|  |  | Archaeplastida | Chlorophyta | Trebouxiophyceae | Trebouxiophyceae_X | Trebouxiophyceae_XX | control |
|  |  | Hacrobia | Telonemia | Telonemia_X | Telonemia_XX | Telonemia_Group_2 | control |
|  |  | Rhizaria | Cercozoa | Endomyxa |  |  | control |
|  |  | Rhizaria | Cercozoa | Endomyxa | Vampyrellida |  | control |
|  |  | Rhizaria | Cercozoa | Endomyxa | Vampyrellida | Leptophryidae | control |
| Autumn | Red soil | Rhizaria | Cercozoa | Endomyxa | Vampyrellida | Vampyrellidae | control |
|  |  | Rhizaria | Radiolaria | Polycystinea |  |  | NS |
|  |  | Rhizaria | Radiolaria | Polycystinea | Spumellarida |  | NS |
|  |  | Rhizaria | Radiolaria | Polycystinea | Spumellarida | Spumellarida_ Group_ I | NS |
|  |  | Stramenopiles | Stramenopiles_X | Bicoecea | Bicoecea_X |  | control |
|  |  | Stramenopiles | Stramenopiles_X | Bicoecea | Bicoecea_X | Bicoecea_XX | control |

**Table S6** Spearman correlations between phylogenetic diversity of microbiomes and physicochemical properties in soils

|  |  | pH | OM | TC | TN | C/N | Moisture | DOC | NO_3_^-^ -N |
| --- | --- | --- | --- | --- | --- | --- | --- | --- | --- |
| Phylogenetic diversity of bacterial community | *R* | 0.514** | -0.390** | -0.515** | -0.520** | 0.445** | -0.471** | -0.515** | -0.146 |
|  | *P* | 0.000 | 0.004 | 0.000 | 0.000 | 0.001 | 0.000 | 0.000 | 0.293 |
| Phylogenetic diversity of fungal community | *R* | -0.369** | 0.250 | -0.119 | 0.378** | -0.392** | 0.261 | 0.371** | -0.114 |
|  | *P* | 0.006 | 0.069 | 0.392 | 0.005 | 0.003 | 0.057 | 0.006 | 0.412 |
| Phylogenetic diversity of protist community | *R* | 0.001 | 0.393** | 0.251 | -0.100 | 0.168 | 0.374** | -0.082 | -0.600** |
|  | *P* | 0.993 | 0.003 | 0.068 | 0.473 | 0.224 | 0.005 | 0.558 | 0.000 |

Abbreviation: OM: organic matter; TC: total content of carbon; TN: total content of nitrogen; C/N: the ratio of TC and TN; Moisture: soil water content; DOC: dissolve organic carbon;

**: *P* < 0.01

**Table S7** Node information of edges appearing in at least two networks linking protist to bacterial or fungal taxa in Fig.3

| Edge id | The edge belongs to the network | Node id | Kingdom | Phylum | Class | Order | Family |
| --- | --- | --- | --- | --- | --- | --- | --- |
| P193-B696 | Black soil_control,  Fluvo-aquic soil_control | P193 | Protist | Cercozoa | Filosa-Imbricatea | Euglyphida | Euglyphidae |
|  |  | B696 | Bacteria | Verrucomicrobia | OPB35 soil group | uncultured bacterium | uncultured bacterium |
| P127-B321 | Black soil_control,  Red soil_control | P127 | Protist | Chlorophyta | Trebouxiophyceae | Chlorellales | Chlorellales_X |
|  |  | B321 | Bacteria | Elusimicrobia | Elusimicrobia | Lineage IIa | uncultured bacterium |
| P130-B321 | Black soil_control,  Red soil_control | P130 | Protist | Chlorophyta | Trebouxiophyceae | Trebouxiophyceae_X | Trebouxiophyceae_XX |
|  |  | B321 | Bacteria | Elusimicrobia | Elusimicrobia | Lineage IIa | uncultured bacterium |
| P127-F106 | Fluvo-aquic soil_control,  Red soil_control | P127 | Protist | Chlorophyta | Trebouxiophyceae | Chlorellales | Chlorellales_X |
|  |  | F106 | Fungi | Basidiomycota | Tremellomycetes | Tremellomycetes_X | Tremellomycetes_XX |
| P120-F5 | Fluvo-aquic soil_control,  Red soil_control | P120 | Protist | Chlorophyta | Chlorophyceae | Chlamydomonadales | Chlamydomonadales_X |
|  |  | F5 | Fungi | Ascomycota | Dothideomycetes | Capnodiales | Mycosphaerellaceae |
| P215-F14 | Black soil_nitrogen addition,  Red soil_nitrogen addition | P215 | Protist | Cercozoa | Filosa-Sarcomonadea | Glissomonadida | Sandonidae |
|  |  | F14 | Fungi | Ascomycota | Eurotiomycetes | Chaetothyriales | Chaetothyriales_X |
| P154-B84 | Black soil_control,  Black soil_nitrogen addition | P154 | Protist | Choanoflagellida | Choanoflagellatea | Acanthoecida | Acanthoecida_X |
|  |  | B84 | Bacteria | Actinobacteria | Actinobacteria | Catenulisporales | Catenulisporaceae |
| P100-F84 | Black soil_nitrogen addition,  Black soil_nitrogen+straw addition | P100 | Protist | Lobosa | Lobosa_X | Lobosa_XX | LKM74-lineage |
|  |  | F84 | Fungi | Basidiomycota | Agaricomycetes | Trechisporales | Hydnodontaceae |

**Table S8** Topological indices used in this study for each network in Fig. S4

|  | Summer | | | Autumn | | |
| --- | --- | --- | --- | --- | --- | --- |
|  | Black soil | Fluvo-aquic soil | Red soil | Black soil | Fluvo-aquic soil | Red soil |
| Clustering coefficient | 0.196 | 0.361 | 0.266 | 0.186 | 0.264 | 0.221 |
| Network density | 0.012 | 0.053 | 0.01 | 0.011 | 0.014 | 0.01 |
| Number of nodes | 200 | 222 | 320 | 223 | 241 | 266 |
| Number of edges | 248 | 1292 | 507 | 270 | 415 | 341 |
| Percentage of nodes assigned to protist taxa (%) | 18.00 | 7.66 | 14.06 | 27.35 | 18.67 | 13.91 |
| Percentage of nodes assigned to fungi taxa (%) | 8.50 | 5.41 | 15.31 | 13.45 | 14.94 | 15.04 |
| Percentage of nodes assigned to bacteria taxa (%) | 73.50 | 86.94 | 70.63 | 59.19 | 66.39 | 71.05 |
| Percentage of edges linked protist taxa to bacterial taxa (%) | 4.03 | 0.23 | 1.18 | 10.37 | 1.45 | 3.52 |
| Percentage of edges linked protist taxa to fungal taxa (%) | 1.61 | 0.39 | 1.18 | 3.33 | 2.89 | 1.76 |
| Percentage of edges linked bacterial taxa to fungal taxa (%) | 4.84 | 0.23 | 7.50 | 6.30 | 3.86 | 6.74 |

**Table S9** Nodes information of edges appearing in at least two networks linking protist to bacterial or fungal taxa in Fig. S4

| Key node id | The edge belongs to the network | Edge id | Node id | Kingdom | Phylum | Class | Order | Family |
| --- | --- | --- | --- | --- | --- | --- | --- | --- |
| P192 |  |  | P192 | Protist | Cercozoa | Filosa-Imbricatea | Euglyphida | Euglyphida_X |
|  | Black soil during summer | P192-F171 | F171 | Fungi | Zygomycota | Zygomycota_X | Zoopagales | uncultured fungus |
|  | Red soil during autumn | P192-F108 | F108 | Fungi | Basidiomycota | Wallemiomycetes | Geminibasidiales | Geminibasidiaceae |
| P193 |  |  | P193 | Protist | Cercozoa | Filosa-Imbricatea | Euglyphida | Euglyphidae |
|  | Black soil during summer | P193-B103 | B103 | Bacteria | Actinobacteria | Actinobacteria | Micrococcales | Microbacteriaceae |
|  | Black soil during autumn | P193-B106 | B106 | Bacteria | Actinobacteria | Actinobacteria | Micromonosporales | Micromonosporaceae |
|  | Black soil during autumn | P193-B370 | B370 | Bacteria | Gemmatimonadetes | Gemmatimonadetes | Gemmatimonadales | Gemmatimonadaceae |
|  | Black soil during autumn | P193-B485 | B485 | Bacteria | Proteobacteria | Alphaproteobacteria | Rhizobiales | KF-JG30-B3 |
|  | Black soil during autumn | P193-B583 | B583 | Bacteria | Proteobacteria | Deltaproteobacteria | Myxococcales | 27F-1492R |
|  | Black soil during autumn | P193-B587 | B587 | Bacteria | Proteobacteria | Deltaproteobacteria | Myxococcales | Cystobacteraceae |
|  | Black soil during autumn | P193-F145 | F145 | Fungi | Glomeromycota | Glomeromycetes | Archaeosporales | Archaeosporales_X |
|  | Black soil during autumn | P193-F51 | F51 | Fungi | Ascomycota | Sordariomycetes | Hypocreales | Bionectriaceae |
|  | Red soil during summer | P193-B165 | B165 | Bacteria | Bacteroidetes | Cytophagia | Cytophagales | Cytophagaceae |
|  | Red soil during summer | P193-B585 | B585 | Bacteria | Proteobacteria | Deltaproteobacteria | Myxococcales | BIrii41 |
| P206 |  |  | P206 | Protist | Cercozoa | Filosa-Sarcomonadea | Cercomonadida | Paracercomonadidae |
|  | Red soil during autumn | P206-B591 | B591 | Bacteria | Proteobacteria | Deltaproteobacteria | Myxococcales | Haliangiaceae |
|  | Black soil during autumn | P206-B435 | B435 | Bacteria | Planctomycetes | Phycisphaerae | WD2101 soil group | planctomycete WY108 |
|  | Fluvo-aquic soil during autumn | P206-F56 | F56 | Fungi | Ascomycota | Sordariomycetes | Microascales | Microascales_X |
|  | Red soil during autumn | P206-B408 | B408 | Bacteria | Parcubacteria | uncultured organism | uncultured organism | uncultured organism |
| P215 |  |  | P215 | Protist | Cercozoa | Filosa-Sarcomonadea | Glissomonadida | Sandonidae |
|  | Black soil during summer | P215-B535 | B535 | Bacteria | Proteobacteria | Betaproteobacteria | Burkholderiales | Burkholderiaceae |
|  | Red soil during summer | P215-B145 | B145 | Bacteria | Armatimonadetes | Ambiguous_taxa | Ambiguous_taxa | Ambiguous_taxa |
|  | Red soil during summer | P215-B307 | B307 | Bacteria | Cyanobacteria | ML635J-21 | uncultured bacterium | uncultured bacterium |
|  | Red soil during autumn | P215-F21 | F21 | Fungi | Ascomycota | Eurotiomycetes | Onygenales | Gymnoascaceae |
|  | Red soil during autumn | P215-F36 | F36 | Fungi | Ascomycota | Pezizomycetes | Pezizales | Karstenellaceae |
| P272 |  |  | P272 | Protist | Stramenopiles_X | Oomycota | Oomycota_X | Oomycota_XX |
|  | Black soil during summer | P272-B486 | B486 | Bacteria | Proteobacteria | Alphaproteobacteria | Rhizobiales | MNG7 |
|  | Black soil during autumn | P272-F37 | F37 | Fungi | Ascomycota | Pezizomycetes | Pezizales | Morchellaceae |
| P204 |  |  | P204 | Protist | Cercozoa | Filosa-Imbricatea | Thaumatomonadida | Thaumatomonadidae |
|  | Black soil during summer | P204-B578 | B578 | Bacteria | Proteobacteria | Deltaproteobacteria | GR-WP33-30 | uncultured delta proteobacterium |
|  | Black soil during autumn | P204-B179 | B179 | Bacteria | Bacteroidetes | Sphingobacteriia | Sphingobacteriales | Sphingobacteriaceae |
|  | Fluvo-aquic soil during summer | P204-F81 | F81 | Fungi | Basidiomycota | Agaricomycetes | Phallales | Phallaceae |

**Table S10** The taxonomic information of protist nodes in Table 5

| Node ID | Kingdom | Phylum | Class | Order | Family | |
| --- | --- | --- | --- | --- | --- | --- |
| P154 | Protist | Choanoflagellida | Choanoflagellatea | Acanthoecida | Acanthoecida_X | |
| P235 | Protist | Ochrophyta | Bacillariophyta | Bacillariophyta_X | Raphid-pennate | |
| P185 | Protist | Cercozoa | Filosa-Granofilosea | Cryptofilida | Mesofilidae | |
| P209 | Protist | Cercozoa | Filosa-Sarcomonadea | Glissomonadida | Allapsidae |  |
| P130 | Protist | Chlorophyta | Trebouxiophyceae | Trebouxiophyceae_X | Trebouxiophyceae_XX |  |

**Reference**

1. Bates ST, Berg-Lyons D, Caporaso JG, Walters WA, Knight R, Fierer N. Examining the global distribution of dominant archaeal populations in soil. ISME J. 2011; 5:908-917.

2. Rousk J, Baath E, Brookes PC, Lauber CL, Lozupone C, Caporaso JG *et al*. Soil bacterial and fungal communities across a pH gradient in an arable soil. ISME J. 2010; 4:1340-1351.

3. Stoeck T, Bass D, Nebel M, Christen R, Jones MD, Breiner HW *et al*. Multiple marker parallel tag environmental DNA sequencing reveals a highly complex eukaryotic community in marine anoxic water. Mol Ecol. 2010; 19 Suppl 1:21-31.
