# Supplementary material for: Protist communities are more sensitive to nitrogen fertilization than other microorganisms in diverse agricultural soils
Source: Microbiome. 2019 Feb 27;7:33. doi: 10.1186/s40168-019-0647-0 (PMC6393985; doi:10.1186/s40168-019-0647-0)
Supplement: Supplementary file 2 — Details regarding network analyses to assess seasonal dynamics of soil microbiome associations and SEM to determine the importance abiotic factors and biotic factors on the significant reductions in the alpha diversity of the protist community. (DOCX 39 kb) [file 40168_2019_647_MOESM2_ESM.docx]

**Additional file 2: Details regarding network analyses to assess seasonal dynamics of soil microbiome associations, and SEM to determine the importance of abiotic and biotic factors on the significant reductions in the alpha diversity of the protist community.**

**Methods:**

**Network analysis**

In order to reveal the effects of seasonal variances on soil microbiome associations in the three soil types, the underlying co-occurrences/associations among protist, bacterial and fungal taxa were depicted through network analyses using the CoNet plug-in in Cytoscape [1, 2]. Data filtering was performed prior to network construction to avoid zero values that could result in spurious correlations and to mitigate the variances of nitrogen fertilization effects, and therefore taxa detected in less than two-thirds of all samples and the sum of taxa counts in all samples less than 10 were discarded [3]. Only those correlations that were significant (*P* < 0.05) by at least three correlation methods were reserved to construct the final network [1, 5]. The other analysis procedures were same as in the description of network analyses in the main text. Furthermore, in order to investigate associations between protists and other microorganisms that can reflect the potential predatory-prey interactions, the protist nodes that linked to the bacterial or fungal taxa appeared in at least two networks were calculated.

**Structural equation model (SEM) construction**

In order to quantify the importance of abiotic factors (nitrogen fertilizers, soil physicochemical properties), and biotic factors (bacterial community, fungal community) on the significant reductions (*P* < 0.05) in the alpha (phylogenetic, faith_pd index) diversity of the protist community in the black and red soil in summer, in the fluvo-aquic and red soil in autumn, SEMs were constructed. Based on current knowledge, the *a priori* and theoretical model was assumed that (i) nitrogen fertilizers could directly influence the alpha diversity of soil protist communities, (ii) nitrogen fertilizers could indirectly affect the alpha diversity of soil protist communities by changing soil physicochemical properties, (iii) nitrogen fertilizers could indirectly influence the alpha diversity of soil protist communities by altering soil bacterial and fungal prey communities (iv) nitrogen fertilizers could indirectly affect soil bacterial and fungal communities by changing soil physicochemical properties. The fertilizers variable was created by assigning the value 0 to the control treatment, 1 to the nitrogen addition treatment, and 2 to the nitrogen plus straw addition treatment. The variances of bacterial and fungal communities were represented by the first NMDS axis based on the unweighted unifrac distance according to the phylogenetic tree at OTU level. Other variables were prepared as described in the main text.

**Results and discussion:**

**Network analysis**

In comparison to summer, the clustering coefficient and network density of microbiome networks in autumn were decreased by 0.051±0.044, 0.013±0.022, respectively, indicating that soil microbiome connections were tighter in summer than in autumn (Fig. S4, Table S8). The percentage of edges linking protist with bacterial or fungal nodes was higher in autumn than in summer in all soil types (bacteria: 3.3±2.69 %; fungi: 1.6±0.97 %; Table S8). We did not detect any consistent links of protist with bacteria or fungi taxa across seasons in any soil type. However, the protist families *Euglyphidae*, *Paracercomonadidae*, *Sandonidae*, *Thaumatomonadidae*, two unknown families within the order Euglyphida and the class Oomycota were identified as potential key protist taxa, as they were found in more than two networks, but associated with different bacterial or fungal taxa depending on soil type or season (Table S9).

Protist diversity and microbiome network complexity was different between summer and autumn, corresponding to large variation in precipitation and soil nitrogen content in two seasons. Therefore, we inferred that soil desiccation and high nitrogen availability drove the seasonal differences in protist diversity, and subsequently changed microbiome associations. This enforces the notion that protists are key controllers of the soil microbiome. However, our results were in contrast with a previous study showing that microbial network became more complex with increased precipitation in grassland soils [9]. These differences might have resulted from the fact that the previous study constructed networks based on the bacterial community alone, while we investigated entire soil microbiomes.

**References**

1. Soffer N, Zaneveld J, Vega Thurber R. Phage-bacteria network analysis and its implication for the understanding of coral disease. Environ Microbiol. 2015; 17:1203-1218.

2. Shannon P, Markiel A, Ozier O, Baliga NS, Wang JT, Ramage D *et al*. Cytoscape: a software environment for integrated models of biomolecular interaction networks. Genome Res. 2003; 13:2498-2504.

3. Hu HW, Wang JT, Li J, Li JJ, Ma YB, Chen D *et al*. Field-based evidence for copper contamination induced changes of antibiotic resistance in agricultural soils. Environ Microbiol. 2016; 18:3896-3909.

4. Hu HW, Wang JT, Li J, Shi XZ, Ma YB, Chen D *et al*. Long-Term Nickel Contamination Increases the Occurrence of Antibiotic Resistance Genes in Agricultural Soils. Environ Sci Technol. 2017; 51:790-800.

5. Steinhauser D, Krall, L. , Müssig, C. , Büssis, D. and Usadel, B. Correlation networks. In: Analysis of Biological Networks. Edited by Y. Pan AYZ, B. H. Junker and F. Schreiber; 2007.

6. Jacomy M, Bastian M, Heymann S. Gephi: an open source software for exploring and manipulating networks. Third International AAAI Conference On Weblogs And Social Media. 2009.

7. Assenov Y, Ramirez F, Schelhorn SE, Lengauer T, Albrecht M. Computing topological parameters of biological networks. Bioinformatics. 2008; 24:282-284.

8. Barberan A, Bates ST, Casamayor EO, Fierer N. Using network analysis to explore co-occurrence patterns in soil microbial communities. ISME J. 2012; 6:343-351.

9. Wang S, Wang X, Han X, Deng Y. Higher precipitation strengthens the microbial interactions in semi-arid grassland soils. Global Ecol Biogeogr. 2018; 27:570-580.
